# Cultivation and molecular characterization of viable *Helicobacter pylori* from the root canal of 170 deciduous teeth of children

**DOI:** 10.1186/s12964-024-01948-5

**Published:** 2024-12-03

**Authors:** Wieland Elger, Nicole Tegtmeyer, Manfred Rohde, Bodo Linz, Christian Hirsch, Steffen Backert

**Affiliations:** 1 Department of Paediatric Dentistry, University School of Dental Medicine, University of Leipzig, Leipzig, Germany; 2 Division of Microbiology, Department Biology, Friedrich-Alexander-Universität Erlangen-Nürnberg, Erlangen, Germany; 3 Central Facility for Microscopy, Helmholtz Centre for Infection Research, Brunswick, Germany

**Keywords:** Endodontics, *Helicobacter pylori*, Root canal, CagA, HtrA, VacA

## Abstract

**Background:**

*Helicobacter pylori* is a persistent pathogen in the human stomach. However, the proposed transmission route via the oral cavity is not understood and under intense debate. While dozens of studies have shown by PCR that *H. pylori* DNA is frequently present in the oral cavity, data on the growth and characterization of viable *H. pylori* from this compartment are very scarce, and it is unclear whether the bacteria can survive in the oral cavity for longer time periods or even colonize it.

**Methods:**

Selective growth methods, scanning electron microscopy, urease assay, Western blotting, PCR, and gene sequencing were applied to identify and examine viable *H. pylori* in decayed milk teeth.

**Results:**

Here, we studied viable *H. pylori* in the plaque and root canals of 170 endodontically infected deciduous teeth that were extracted from 54 children*.* While *H. pylori* DNA was detected in several plaque and many root canal samples by PCR, live bacteria could only be cultivated from 28 root canals, but not from plaque. These 28 isolates have been identified as *H. pylori* by PCR and sequencing of *vacA, cagA* and *htrA* genes, phylogenetic analyses, protein expression of major *H. pylori* virulence factors, and by signal transduction events during infection of human cell lines.

**Conclusions:**

Thus, the microaerobic environment in the root canals of endodontically infected teeth may represent a protected and transient reservoir for live *H. pylori*, especially in individuals with poor dental hygiene, which could serve as a potential source for re-infection of the stomach after antibiotic therapy or for transmission to other individuals.

**Supplementary Information:**

The online version contains supplementary material available at 10.1186/s12964-024-01948-5.

## Background

*Helicobacter pylori* is a microaerobic Gram-negative bacterium that infects worldwide about 4.4 billion people, which corresponds to approximately half of the global population [1]. *H. pylori* is a model organism for persistent microbial infection and chronic inflammation. The pathogen specifically colonizes the antrum of the human stomach, and is involved in the onset of chronic gastritis, peptic ulceration and malignant diseases such as mucosa-associated lymphoid tissue (MALT) lymphoma and gastric adenocarcinoma [2]. However, the majority of individuals infected by *H. pylori* remains asymptomatic. Only ~ 10–15% of the colonized persons develop peptic ulcer disease, and ~ 1–2% develop malignant changes [3]. Due to the causal link with malignancies, the International Agency for Research on Cancer categorized *H. pylori* as a type I carcinogen [4]. The GLOBOCAN statistics report for the year 2020 described 1,089,103 new cases of gastric cancer, which corresponds to ~ 5.7% of all human cancers worldwide [5]. In total, these cases are responsible for 769,793 deaths per year, which commonly occur late in life [5] and make stomach cancer a frequent type of cancer in humans. Conversely, colonization by *H. pylori* early in life can be favorable for its human host by providing a remarkable protection against allergic diseases such as asthma, inflammatory bowel disease (IBD) and gastroesophageal diseases through manipulation of the human immune system [6, 7].

*H. pylori* infection of the stomach and gastric disease development depend on numerous microbial factors. The bacteria survive at very low pH in the gastric environment by employing its urease enzyme to maintain an almost neutral pH in the cytoplasm. Thus, the urease complex is fundamental to *H. pylori* survival and virulence [8]. A major *H. pylori* virulence factor is the cytotoxin-associated gene (*cag*) pathogenicity island (PAI) that encodes ~ 30 Cag proteins, which assemble to form a type-IV secretion system (T4SS) [9, 10]. The *cag*PAI is typically present in highly virulent type-I *H. pylori* strains, and absent in less virulent type-II strains [11, 12]. This T4SS translocates the effector protein CagA as well as the lipopolysaccharide component ADP-glycero-β-D-*manno*-heptose (ADPH) into gastric epithelial cells [13]. These T4SS functions are mediated by binding of the non-*cag*PAI adhesin outer membrane protein Q (HopQ) to carcinoembryonic antigen-related cell adhesion molecule (CEACAM) receptors [14, 15]. Upon delivery, CagA undergoes tyrosine-phosphorylation at EPIYA-motifs (named A, B and C in Western strains and A, B and D in East Asian strains) by the host kinases Src and Abl [16–18]. Subsequently, translocated CagA activates intracellular signaling cascades that influence the dynamics of the actin cytoskeleton, cell motility via phosphorylation of cortactin, cell proliferation via activation of β-catenin and various other pathways [19, 20]. On the other hand, injected ADPH stimulates the activation of pro-inflammatory transcription factor NF-κB and interleukin-8 (IL-8) release [21–24]. Moreover, it was shown that the T4SS core components and pilus-associated proteins CagL and CagY can bind and activate toll-like receptor 5 (TLR5), further activating crucial innate immune responses by *H. pylori* [25, 26]. In addition, the intracellular nucleic acid receptor TLR9 was shown to be activated by the *cag* T4SS, but the proposed translocated DNA substrate is still unknown [27–30]. Another virulence factor is the *H. pylori* vacuolating cytotoxin A (VacA) that produces large cellular vacuoles and triggers apoptosis in infected epithelial cells [31, 32]. This VacA activity depends on specific alleles present in different *H. pylori* strains, with the highly cytotoxic *s1/m1* allele, less toxic *s1/m2* and non-toxic *s2/m2* alleles being described [12, 31, 33]. Finally, *H. pylori* employs the serine protease HtrA to disrupt gastric epithelial barrier functions [34]. Functional analyses discovered a single leucine/serine (L/S) polymorphism at HtrA position 171 that affects the stability of proteolytically active trimers and regulates processing of epithelial cell junctional proteins upon infection [35]. 171L-type HtrA, but not 171S-type HtrA strains, were shown to trigger increased cleavage of occludin and E-cadherin, and enhanced chromosomal DNA damage, which in combination cause malignant changes in infected gastric epithelial cells.

Humans are the only known natural host of *H. pylori*; environmental reservoirs of this bacterium outside of the human stomach are unknown. Thus, transmission of the microbe is believed to occur directly between human individuals, typically early in childhood and among family members [36, 37]. The three major reported routes – oral-to-oral, gastric-to-oral and fecal-to-oral – are under controversial discussion [36, 38–40]. Reports on the isolation and characterization of viable *H. pylori* from the oral cavity are rare. In one study, viable *H. pylori* were commonly cultured from vomit, and sporadically from saliva samples and diarrhoeic stool of *H. pylori*-positive patients [39]. Therefore, it has been proposed that *H. pylori* may be transmitted during the occurrence of gastrointestinal disease, especially by vomiting [39]. Occasionally, *H. pylori* DNA was also detected in the mouth, usually from samples of dental plaque, saliva and oral mucosa [37, 38, 40]. However, in many studies the bacteria were only investigated by indirect detection methods, for example by PCR, RT-PCR, ELISA, multilocus sequence typing (MLST), urease test and others, but definitive proof for *H. pylori* was often missing [41–48]. Some researchers have even expressed strong doubts on previous studies if live *H. pylori* were actually ever isolated from plaque, tongue or saliva [38]. Given that data on the detection and unambiguous characterization of live and viable *H. pylori* are largely missing and/or inconclusive, studies with large numbers of patients are required to end this controversy.

We previously detected viable *H. pylori* in the infected root canals of deciduous teeth of three children [49]. In the present study, we have enlarged the patient cohort and analyzed a total of 170 teeth and dental plaque samples from 54 children. We successfully cultured 28 viable *H. pylori* strains from the root canal of these endodontic-infected deciduous teeth and applied a combination of multiple methods for a detailed characterization of the strains at the molecular level.

## Materials and methods

### Patient selection and teeth extraction

Patient selection and teeth extraction was conducted at the Department of Paediatric Dentistry and Primary Prevention at the University Hospital of Leipzig from July 2022 to August 2023. Written informed consent was obtained from the patients' parents regarding the planned extraction of primary teeth and the medical necessity of the procedure. The indication for extraction included only non-salvageable, endodontically infected deciduous teeth. The study protocol was approved by the Ethics Committee of the University of Leipzig under the number 019/13-ff. A total of 54 patients (30 boys and 24 girls aged 1 to 12 years with a mean age of 6.22 years) participated in the study. Tooth extractions were performed under both general anaesthesia and local anaesthesia in the dental chair, with 1 to 9 teeth extracted per patient, with a total of 170 teeth. On average, 3 teeth were extracted per patient, sometimes over several sessions. After extraction with the appropriate dental forceps, the teeth were immediately transferred to sterile reaction tubes filled with brain heart infusion (BHI) medium (Oxoid, Wesel, Germany) and 20% glycerol for storage without any further pre-treatment or alteration of the teeth. The samples were intermediately stored in a dedicated refrigerator at -80 °C to maintain the bacteria in a stable state until further analysis. Each tube was numbered to ensure confidentiality of patient data. The numbering allowed only the dental team to infer the identity of the patients, and no external person had access to this information.

### *H. pylori* growth and DNA isolation

After the initial storage at -80 °C, two samples were taken from each tooth: a supragingival plaque sample and a root canal sample. For this purpose, the supragingival plaque was scraped off the teeth using a sterile scalpel. Then, the teeth were broken open with sterile pliers and the content from root canals was scraped off using a second sterile scalpel. These samples were homogenized and subsequently split for three different analyses, namely the isolation of genomic DNA, electron microscopy, and *H. pylori* cultivation. DNA isolation was performed using a commercial kit according to the manufacturer's instructions (Qiagen, Hilden, Germany). For cultivation, the samples were plated on GC agar plates, supplemented with 10% horse serum (Pan Biotech, Aidenbach, Germany), 1% vitamin mix and antibiotics (10 μg/ml colistin, 10 μg/ml nystatin, 5 μg/ml trimetroprim, and 10 μg/ml vancomycin, all from Sigma-Aldrich (St. Louis, MO, USA). The plates were incubated at 37 °C for 7 days in an anaerobic jar containing a CampyGen gas-generation sachet (Oxoid) to create a microaerobic atmosphere. The *H. pylori* control strain 26695 [50] and its isogenic *ureB* deletion mutant (26695Δ*ureB*) were cultured from frozen stocks. To enable selection for the *ureB* mutant strain, chloramphenicol (6 µg/mL, Sigma-Aldrich) was added to the media.

### Functional urease test

To select bacteria that produce functional urease, individual colonies were streaked onto GC agar plates containing urea (600 µg/mL) and the color indicator phenol red (100 µg/mL). The medium was acidified to pH 5 as described [51].

### Field emission scanning electron microscopy

For field-emission scanning electron microscopy, small tooth fragments from three independent preparations as well as cultured *H. pylori* and plaque bacteria were fixed in 5% formaldehyde and 2% glutaraldehyde in cacodylate buffer (0.1 M cacodylat, 0.01 M CaCl_2_, 0.01 M MgCl_2_, 0.09 M saccharose, pH 6.9). Samples were centrifuged and washed twice with TE buffer (10 mM TRIS, 2 mM EDTA, pH 6,9). Dehydration was achieved with a graded series of ethanol (10, 30, 50, 70, 90%) on ice for 10 min for each step, followed by two steps with 100% ethanol (stored over CuSO_4_) for 15 min each step. Further dehydration was carried out with tetramethylsilan (TMS) according to the following scheme: 1 part ethanol:1 part TMS, 1 part ethanol:2 parts TMS, 1 part ethanol:3 parts TMS and 1 part ethanol:4 parts TMS, before further processing twice in TMS alone for 5 min at room temperature. After air drying samples were attached to stubs with adhesive carbon tape and sputter coated with gold/palladium (Bal-Tec SCD500) and examined in a Zeiss field emission scanning electron microscope DSM982 at an acceleration voltage of 5 kV using the Everhart–Thornley SE detector.

### Cultivation and infection of wild-type AGS cells and AGS cells expressing E-cadherin

For infection experiments, the epithelial cell line AGS (ATCC #CRL-1739™) was used, which was originally isolated from the stomach tissue of a patient with gastric adenocarcinoma. Cells were routinely cultured in RPMI 1640 medium with 10% fetal bovine serum (FBS, both Thermo Fisher Scientific Waltham, MA, USA) and antibiotics (1% penicillin/streptomycin purchased from Sigma-Aldrich and 0.2% Normocin™ antimicrobial reagent from InvivoGen, Toulouse, France). The cultivation conditions in the incubator were set to 37 °C and 5% CO_2_ in a humidified atmosphere. Regular AGS wild-type cells are deficient in E-cadherin. AGS cells were stably transduced with wild-type human E-cadherin encoded by a lentiviral plasmid to obtain polarized AGS-E-cadherin cells [52]. Immunofluorescence microscopy and/or Western blotting were applied to confirm the expression of cell-to-cell junction proteins such as E-cadherin, p120-catenin, α-catenin and β-catenin in this cell line [52, 53]. Prior to infection of these cells, the growth medium was removed by washing the cells twice with phosphate-buffered saline (PBS, Sigma-Aldrich) and replaced with fresh medium without the addition of antibiotics. The cells were then seeded into 6-well plates and grown to 70–80% confluence before being infected with *H. pylori* isolates from root canal samples. Infections were performed at a multiplicity of infection (MOI) of 50 for 6 to 24 h, depending on the assay, and were then harvested and processed.

### Secreted Embryonic Alkaline Phosphatase (SEAP) reporter assays

For analyzing the induction of transcription factor NF-κB by *H. pylori*, AGS cells were grown to confluence in 6-well plates followed by transfection with 5 µg of the pNF-κB-SEAP reporter plasmid for 48 h (http://www.addgene.org) by using TurboFect™ transfection reagent (Thermo Fisher Scientific) [54]. To monitor toll-like receptor-5 (TLR5) and TLR9 activation by *H. pylori,* we used the human TLR5/NF-κB/SEAP reporter HEK293 cells (HEK-Blue-hTLR5), TLR9/NF-κB/SEAP reporter HEK293 cells (HEK-Blue-hTLR9), and HEK-Blue-Null1 cells (all three from Invivogen) as control [25, 55]. These three cell lines were infected with the *H. pylori* isolates cultivated from root canal samples at MOI of 50 for 24 h. NF-κB-dependent SEAP production was quantified in 96-well plates by dispensing 180 μL of QUANTI-Blue™ solution (InvivoGen) per well and adding 20 μL of the infected or non-infected control cell culture supernatant to each well. After a 30 min incubation at 37 °C, the optical density (OD) was measured at 620 nm using a microplate reader (Infinite F200 Pro, Tecan, Grödig, Austria).

### Luciferase reporter assay for β-catenin

The Wnt/β-catenin signaling pathway activation was studied using the TCF Reporter Plasmid Kit (Sigma-Aldrich) containing two transfection grade T cell factor (TCF) reporter gene plasmids, TOPFlash and FOPFlash, for luciferase reporter assays**.** TOPflash is a firefly luciferase reporter plasmid that responds to β-catenin activation, harboring functional binding sites for TCF/lymphoid enhancer factor (LEF) transcription factors upstream of the thymidine kinase minimal promoter and the luciferase open reading frame. In contrast, FOPflash contains mutated TCF binding sites, so that no or only reduced expression of the reporter gene can occur and serves as a negative control for TOPflash activity. AGS cells were transfected with TOPflash or FOPflash using TurboFect™ transfection reagent according to the manufacturer's protocols. 48 h after transfection, cells were infected with the *H. pylori* isolates from root canal samples at MOI of 50 for 24 h. The cells were then lysed and analyzed for firefly luciferase activity by using an Orion microplate luminometer (Berthold, Germany).

### Protein profiling, SDS-PAGE and immunoblotting

Protein profiles were obtained from bacterial isolates grown on plates. For this purpose bacterial pellets were lysed in hot SDS buffer for 10 min, loaded onto 8–12% SDS-PAGE gels and analyzed by Coomassie brilliant blue staining or immunoblotting. To examine the expression of Urease B, VacA and HtrA, the separated proteins were blotted onto a polyvinylidene difluoride (PVDF) membrane (Carl Roth, Karlsruhe, Germany) and were analyzed with the following primary antibodies: rabbit α-Urease B using the conserved peptide HDYTIYGEELK as antigen (#21,987, BioGenes GmbH, Berlin, Germany) [56], rabbit α-VacA (kindly provided by Dr. Timothy Cover, Vanderbilt University, Nashville, USA) [57, 58] and rabbit α-HtrA against the entire recombinant protein (#26823, BioGenes GmbH) [59]. Samples from infected AGS cells were further prepared for Western Blot analysis using mouse α-PY99 (#sc-7020, Santa Cruz Biotechnology, Dallas, TX, USA), rabbit α-CagA (#HPP-5003–9, Austral Biologicals), rabbit α-JAM-A (#ab52647, Abcam, Cambridge, UK), rabbit α-HopQ (a kind gift from Prof. Markus Gerhard, Technische Universität München, Munich, Germany) and rabbit α-CagY and α-CagM (raised against the recombinant proteins, BioGenes GmbH) antibodies. The α-phospho-CagA-EPIYA-C antibody was described in [60]. Goat α-rabbit and goat α-mouse IgGs conjugated to horseradish peroxidase (HRP) (both Thermo Fisher Scientific) were applied as secondary antibodies [61]. Chemiluminescence was detected using an ECL mixture of 1.41 mM luminol in 0.1 M Tris–HCL (pH 8.6) together with 0.61 mM p-coumaric acid in DMSO and 0.02% hydrogen peroxide and visualized with a ChemiDoc XRS + Gel Imaging System (Bio-Rad, Hercules, CA, USA) [62].

### Dotblot assay

Ten μg purified total proteins from *H. pylori-*infected AGS cells were prepared and subsequently immobilized on Protran® nitrocellulose transfer membranes (Whatman, Maidstone, Kent, UK) employing the BioDot SF system of BioRad (Munich/Germany) as reported earlier [25, 26]. Virulence protein expression was assessed using the antibodies and protocol described above.

### Genotyping of *cagA* and *vacA* genes by PCR

The number and types of EPIYA-motifs in *cagA* were detected by polymerase chain reaction (PCR) amplification with different primer pairs as previously described [63]. Each 25 μL PCR reaction mix consisted of: 50 ng of genomic template DNA, 0.4 mM of the forward primer, 0.08 mM of the reverse primer (Eurofins GmbH, Ebersberg, Germany), 0.2 mM each of the dNTPs (Promega, Madison, WI, USA), 1.25 U Taq DNA polymerase and 1 × ThermoPol® reaction buffer (both New England Biolabs, Frankfurt am Main, Germany). PCR reactions were performed in a Primus 96 Thermocycler (Peqlab Biotechnologie GmbH, Erlangen, Germany). For the initial denaturation step, the reaction mixture was heated to 95 °C for 90 s, followed by 35 cycles of denaturation at 95 °C for 30 s, annealing at 57 °C for 60 s, and extension at 72 °C for 30 s. After the 35 cycles, a final extension was carried out at 72 °C for 5 min. All PCR products were detected by gel electrophoresis on a 1% agarose gel stained with an ethidium bromide solution (Carl Roth) and visualized under UV light using the ChemiDoc XRS + Gel Imaging System (Bio-Rad).

To determine the *vacA* subtypes (*s1, s2, m1* and *m2*), the signal sequence region and the middle region of the *vacA* gene were amplified using the described primer pairs [64]. The PCR reactions were carried out in a final volume of 25 μl each, containing 50 ng DNA, 0.5 mM of the forward and reverse primers, 0.2 mM each of the dNTPs, 1.25 U Taq DNA polymerase and 1 × ThermoPol® reaction buffer. The PCR temperature profile was: 94 °C for 9 min, followed by 40 cycles at 94 °C for 30 s, 50 °C for 45 s and 72 °C for 45 s and a final incubation at 72 °C for 5 min. The PCR amplicons were analyzed as described above.

### *htrA* sequence analysis

A 1.3 kb fragment of the *htrA* gene was amplified by PCR using primers 1081F (5 ‘-CAR TAT CCA AAT CCA AAR CAT GCC) and 556R (5 ‘-TCA TTT CAC CAA AAT GAT CCT AT), and Sanger sequencing was performed using primer 610R1 (5 ‘-CAC ACC AAA ATC CCT GC). The resulting 822 bp gene fragments were joined with *htrA* from the genomes of *H. pylori* reference strains 26695, P1, P12, B128, G27, HPAG1, N6, J166, and SouthAfrica7, and from *H. cetorum* strain MIT99-5656. A Neighbor-joining tree was constructed in MEGA version X [65] using the Maximum Composite Likelihood model. The *htrA* sequences were analyzed for S- or L-type variants at amino acid position 171 (171S or 171L, see Table 1) using BLASTX.

**Table 1:** Molecular characteristics of 28 viable *H. pylori* strains isolated from 170 root canals of human teeth

	Expression of bacterial virulence factors***							Host responses upon infection****					
Strain # *	Urease	HopQ	CagY	CagM	HtrA	CagA- EPIYAs	VacA allele	Vacuole formation	Phospho -CagA	Cell elongation	NF-κB activation	β-catenin activation	TLR5/TLR9 activation
SBA-01	+	+	+	+	S-type	ABC	*s1m1*	+ + +	+ +	+ +	+ + +	+	+ + +
SBA-02	+	+	+	+	L-type	ABC	*s1m2*	+	+ +	+ +	+ + +	+	+ + +
SBA-03**	+	+	-	-	S-type	-	*s2m1*	-	-	-	-	-	-
SBA-04	+	+	+	+	L-type	ABC	*s1m1*	+ + +	+ +	+ +	+ + +	+ + +	+ + +
SBA-05	+	+	+	+	S-type	ABCCC	*s1m1*	+ + +	+ + +	+ + +	+ + +	+	+ + +
SBA-06	+	+	-	-	S-type	-	*s2m2*	-	-	-	-	-	-
SBA-07**	+	+	+	+	L-type	ABCC	*s1m1*	+ + +	+ + +	+ + +	+ + +	+ + +	+ + +
SBA-08	+	+	+	+	S-type	ABCC	*s1m1*	+ + +	+ + +	+ + +	+ + +	+	+ + +
SBA-09	+	+	+	+	S-type	ABC	*s1m1*	+ + +	+ +	+ +	+ + +	+	+ + +
SBA-10	+	+	+	+	S-type	ABC	*s1m1*	+ + +	+ +	+ +	+ + +	+	+ + +
SBA-11	+	+	+	+	L-type	ABCC	*s1m1*	+ + +	+ + +	+ + +	+ + +	+ + +	+ + +
SBA-12**	+	+	+	+	S-type	ABCC	*s1m1*	+ + +	+ + +	+ + +	+ + +	+	+ + +
SBA-13	+	+	+	+	S-type	ABC	*s1m1*	+ + +	+ +	+ +	+ + +	+	+ + +
SBA-14	+	+	+	+	L-type	ABCCC	*s1m2*	+	+ + +	+ + +	+ + +	+ + +	+ + +
SBA-15	+	+	+	+	S-type	ABCC	*s1m1*	+ + +	+ + +	+ + +	+ + +	+	+ + +
SBA-16	+	+	-	-	L-type	-	*s2m2*	-	-	-	-	-	-
SBA-17	+	+	+	+	L-type	ABC	*s1m1*	+ + +	+ +	+ +	+ + +	+ + +	+ + +
SBA-18**	+	+	+	+	L-type	ABC	*s1m1*	+ + +	+ +	+ +	+ + +	+ + +	+ + +
SBA-19	+	+	+	+	S-type	ABCC	*s1m1*	+ + +	+ + +	+ + +	+ + +	+ +	+ + +
SBA-20	+	+	+	+	S-type	ABCC	*s1m1*	+ + +	+ + +	+ + +	+ + +	+	+ + +
SBA-21	+	+	+	+	S-type	ABC	*s1m1*	+ + +	+ +	+ +	+ + +	+	+ + +
SBA-22	+	+	-	-	S-type	-	*s1m2*	+	-	-	-	-	-
SBA-23**	+	+	-	-	S-type	-	*s1m1*	+ + +	-	-	-	-	-
SBA-24	+	+	+	+	S-type	ABCCC	*s1m1*	+ + +	+ + +	+ + +	+ + +	+	+ + +
SBA-25	+	+	+	+	S-type	ABCC	*s1m1*	+ + +	+ + +	+ + +	+ + +	+	+ + +
SBA-26	+	+	-	-	S-type	-	*s2m2*	-	-	-	-	-	-
SBA-27	+	+	+	+	L-type	ABCC	*s1m2*	+	+ + +	+ + +	+ + +	+ + +	+ + +
SBA-28	+	+	+	+	S-type	ABCC	*s1m1*	+ + +	+ + +	+ + +	+ + +	+	+ + +

^*^We detected viable *H. pylori* in the indicated 28 teeth, named SBA-1 to SBA-28. In addition, 4 teeth were also positive by *vacA* PCR, but *H. pylori* growth on agar plates was negative (not shown)

^**^In these 5 teeth, plaque samples also revealed a PCR product for the *vacA* gene, but no *H. pylori* growth on agar plates

^***^Expression of urease B, HopQ, CagM, CagY, CagA, phospho-CagA and HtrA by Western blotting (+ , yes; -, no) (compare with Figs. 3, 5 and S1). S- and L-type HtrAs were determined by gene sequencing. Typing of CagA EPIYAs and VacA alleles was done by specific PCRs

^****^ + + + , strong responses; + + , intermediate responses, + , weak responses, -, no responses (compare with Figs. 5 and 6)

### Statistical evaluation

All statistical evaluations were performed using Graph Pad Prism 8.0 (GraphPad Software, San Diego, CA, USA) and were based on data sets from at least three independent experimental trials. For multiple comparisons, the non-parametric Kruskal–Wallis test, followed by Dunn's correction was used. In Fig. 7, the data are represented as box plots, showing the median (line in the box) with the first quartile (bottom of the box) and third quartile (top of the box). The whiskers indicate the minimum and maximum data values. The *p*-values *p* ≤ 0.05 ( ∗) and *p* ≤ 0.01 (∗ ∗) represent a statistically significant result.

## Results

### Identification of *H. pylori* DNA in teeth by PCR

**Fig. 1:**
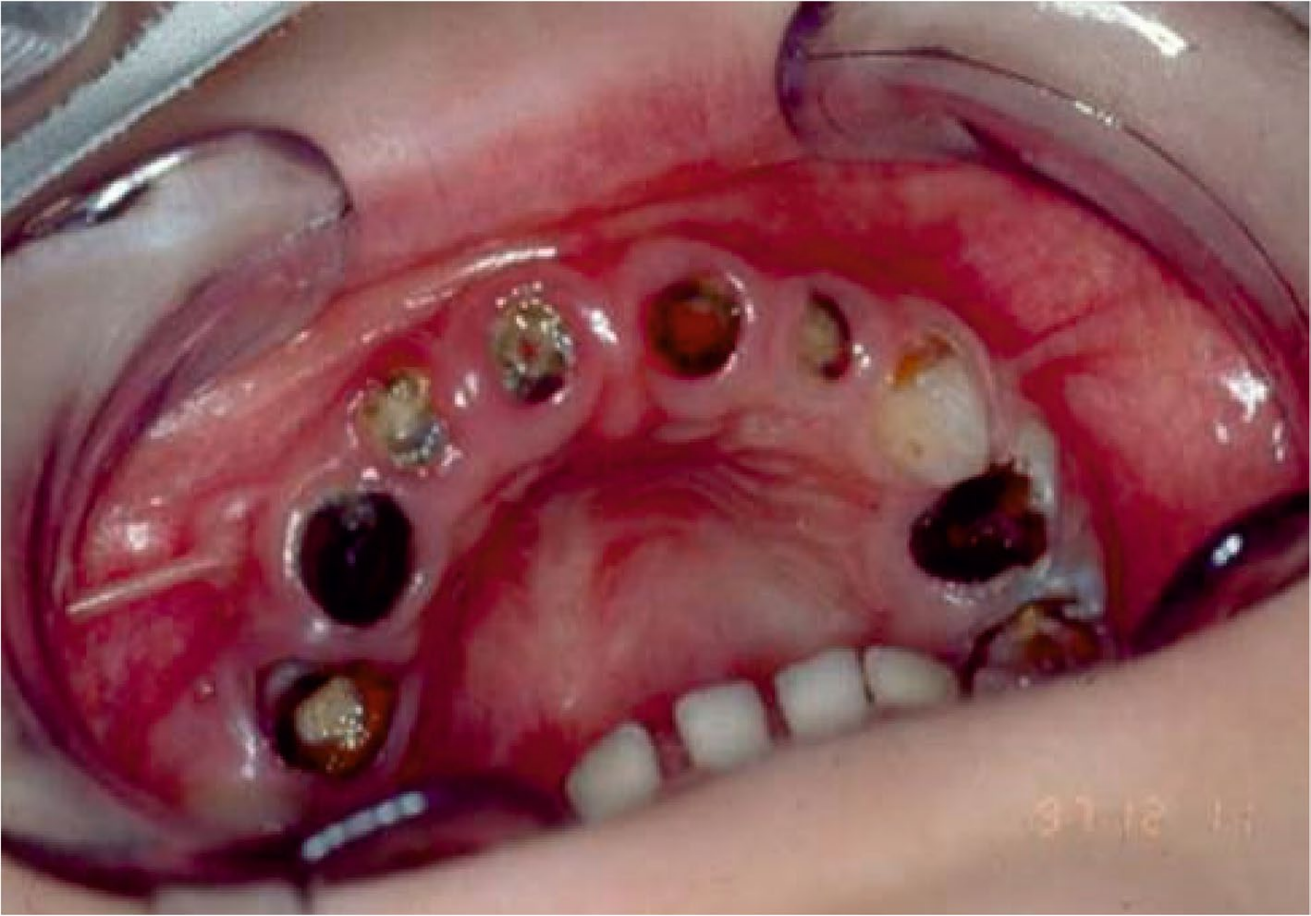
Photograph of the oral cavity of one representative patient in the present study. The picture was taken from a 3.5-year-old child showing severe early childhood caries associated with the lack of oral hygiene and massive dental plaque deposits

To study the presence of *H. pylori* in the oral cavity, we initially extracted teeth from 54 children (Fig. 1) and isolated total DNA from both root canal and plaque samples of 170 teeth. The samples were analyzed by PCR amplification of the *vacA* gene that encodes the vacuolating cytotoxin VacA. *vacA* is a *H. pylori*-specific gene that is present in all strains. Evolutionarily related *vacA* genes are only found in the close relative *Helicobacter cetorum* from the stomachs of dolphins and whales [66, 67], and fragmented *vacA* genes were detected in *Helicobacter acinonychis* colonizing the stomachs of big predator cats [56, 68]. We used *H. pylori*-specific primer sets to amplify the *s1/s2* and *m1/m2* allele regions of *vacA* [64]. We were able to obtain PCR products from 32 root canal and 5 plaque samples (Table 1). These findings suggest that DNA of *H. pylori* can be obtained from some, but not all individuals.

### Visualization of *H. pylori* in teeth by electron microscopy

**Fig. 2:**
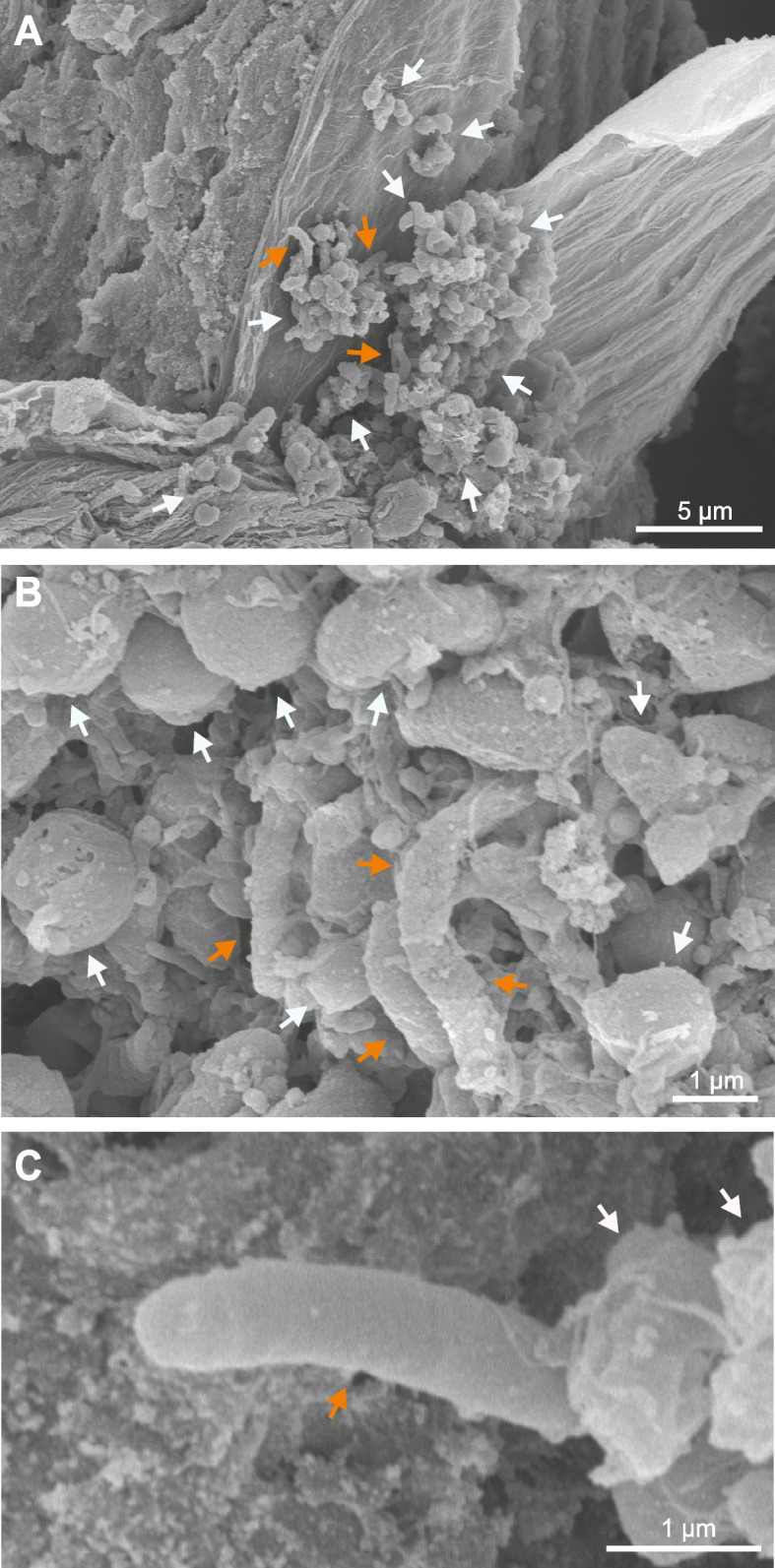
Electron microscopic analysis of root canal samples from teeth. **A**-**C** Field-emission scanning electron microscopy unravelled the presence of various spiral-shaped rod-like microbes that may correspond to *Helicobacter pylori* (orange arrows). These *H. pylori* candidates had a length of approximately 3–4 µm and a diameter of ~ 0.5–0.7 µm. The major portion of detected microbes, however, had a coccoid shape and probably correspond to other Gram-negative or Gram-positive bacteria (white arrows). Representative micrographs from three preparations are shown

In order to visualize characteristic *H. pylori* bacteria in the root canals, the samples were examined by field-emission scanning electron microscopy. The images revealed the presence of numerous spiral-shaped organisms with the typical shape of *H. pylori* that were seen attached to the inner teeth canal surface (Fig. 2A-C, orange arrows). These *H. pylori*-like candidates had a diameter of approximately 0.5–0.7 µm and a length of ~ 3–4 µm. Additionally, the majority of detected bacteria were coccoid forms, probably representing other Gram-negative or Gram-positive bacteria (Fig. 2A, white arrows). This examination advocates the possible occurrence of viable, spiral-shaped *H. pylori*, at least in a subset of root canal samples. These findings revealed no specific association with the age of the patients, their gender and the type and location of each *H. pylori-*positive tooth (Table S1).

### Isolation and characterization of viable *H. pylori* from teeth

**Fig. 3:**
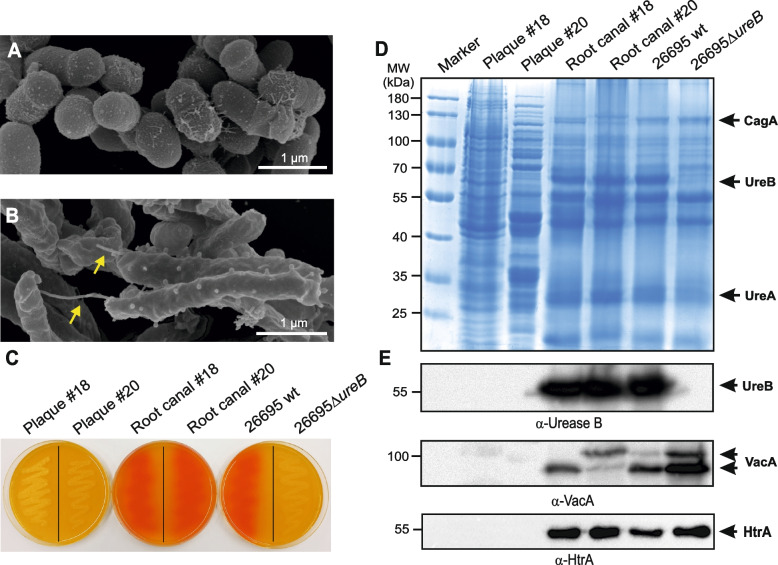
Electron microscopy, urease tests and protein expression analyses of isolated bacterial colonies from teeth samples. Field-emission scanning electron microscopy of (**A**) Gram-positive cocci from plaque and (**B**) Gram-negative rods from root canals of extracted teeth. Yellow arrows indicate bacterial flagella. **C **Bacterial samples from extracted teeth were analysed for the production of urease activity on acidified agar plates containing urea as substrate. Bacterial samples from left to right: plaque (from teeth # 18 and 20), root canal (from teeth # 18 and 20) and control *H. pylori* strains 26695 wt and 26695Δ*ureB* mutant. Growth of bacterial isolates from the root canals and 26695 wt expressing a functional urease triggered a typical colour change (from orange to red), while this colour change was not induced by plaque bacteria or 26695Δ*ureB* mutant, demonstrating that urease activity is absent. **D **Total protein profiling of the grown bacteria shown in (**C**) by SDS-PAGE and Coomassie staining. The gels of root canal samples and 26695 showed the characteristic *H. pylori* banding pattern. The position of *H. pylori* CagA, urease A and urease B (the two major urease subunits) is marked with arrows. **E **Western blot investigation of the above samples using specific antibodies against major *H. pylori* virulence proteins urease B (UreB), vacuolating cytotoxin VacA and serine protease HtrA revealed their presence in root canal samples and *H. pylori* control strain 26695, but not in plaque bacteria. The results are representative from at least three independent experiments

In parallel, all tooth samples (plaque and root canal) were incubated under microaerobic growth conditions for up to eight days using selective GC agar plates to isolate the bacteria [49]. Individual *H. pylori* colonies grew from 28 of 170 root canal samples, suggesting that 16.5% of the teeth were *H. pylori*-growth positive. In contrast, no *H. pylori* colonies were obtained from the plaque samples. Next, the isolated *H. pylori*-like bacteria grown on GC agar plates and bacterial colonies cultured from the corresponding dental plaque were subjected to field-emission scanning electron microscopy. The resulting images revealed typical coccoid Gram-positive bacteria from the plaque (Fig. 3A) and bacteria with the typical *H. pylori* shape from the root canals (Fig. 3B). In the latter samples, we also noted various mono-polar flagella that are characteristic for *H. pylori* bacteria [69].

### Investigation of typical *H. pylori* characteristics

Subsequently, these bacterial colonies were cultivated on acidified GC agar plates containing urea, the natural substrate of the urease enzyme [8]. These experiments showed functional urease activity in the *H. pylori*-like bacteria from root canal samples that was similar to *H. pylori* strain 26695 that was used as positive control. In contrast, retarded growth and no urea hydrolysation was seen from the isogenic Δ*ureB* mutant or in all non-*H. pylori* bacteria from dental plaque (Fig. 3C). To unquestionably identify *H. pylori*, we compared the total protein profile of purified bacteria from plaque and root canal samples with that of wild-type (wt) *H. pylori* strain 26695 and Δ*ureB* mutant. The results from SDS-PAGE show that the protein patterns from the root canal bacteria were highly similar to that of the *H. pylori* control, while those from dental plaque were clearly different as expected for other Gram-negative or Gram-positive bacteria (Fig. 3D). The same protein samples were also subjected to Western blotting that were probed with highly specific *H. pylori* antibodies against urease B, VacA and HtrA. The results show typical bands for *H. pylori* at the expected sizes for urease B (~ 55 kDa), VacA (~ 80 and ~ 90 kDa subunits) and HtrA (~ 55 kDa) (Fig. 3E, lanes 4–7), but not for plaque samples (Fig. 3E, lanes 2–3). As another control, the Δ*ureB* mutant in lane 7 revealed no band for urease B, as expected.

### Sequencing of *H. pylori htrA* genes and phylogenetic analyses

**Fig. 4:**
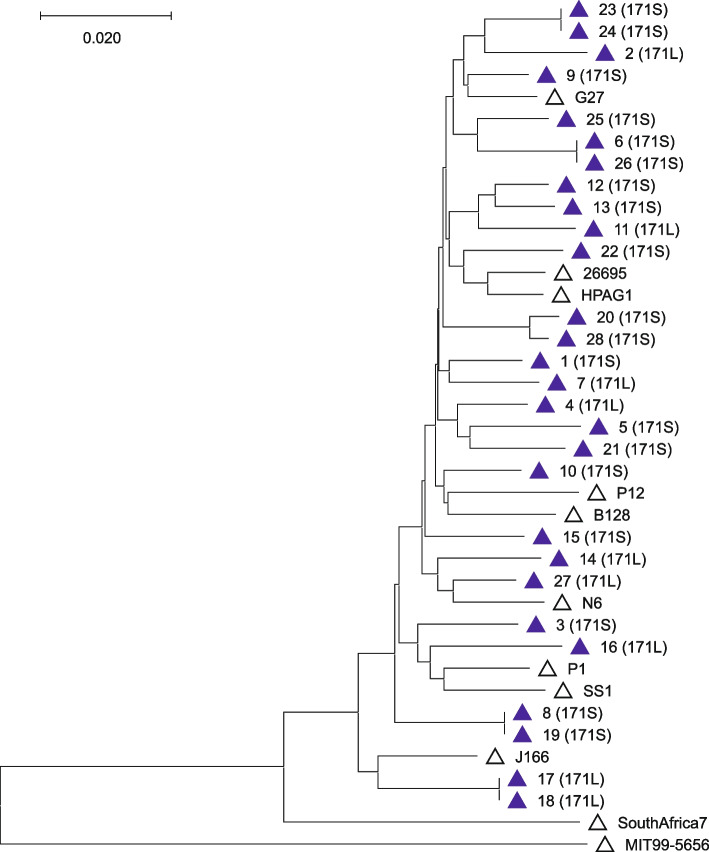
*htrA* gene analysis of *H. pylori* from the root canal of extracted teeth. The *htrA* genes were sequenced from the 28 *H. pylori* strains obtained from the root canal of deciduous teeth. Neighbour joining tree calculated from these *htrA* gene sequences (blue filled triangle) and from *H. pylori* reference genomes (open triangle). The *htrA* gene from *H. cetorum* strain MIT99-5656 was used as an outgroup. The HtrA variant (171S or 171L) of the isolated strains from teeth is indicated

Sequence and Neighbour joining tree analysis of an 822 bp long *htrA* gene fragment revealed the presence of 24 unique haplotypes out of the 28 *htrA* sequences (Fig. 4); four sequences were duplicates (samples 6 + 26; 8 + 19; 17 + 18; 23 + 24). Each of the duplicate sequences originated from additional teeth extracted from the same respective patient. All of the new sequences showed up to 98% similarity to *H. pylori htrA* sequences in Genbank. Eight strains possessed a 171L-type HtrA (Fig. 4, Table 1) that was previously shown to be associated with an increased risk of gastric cancer development [35], while 19 strains had a more benign 171S-type HtrA variant. In the Neighbour joining tree, the *htrA* sequences of *H. pylori* from teeth were interspersed by those from the genome of *H. pylori* reference strains 26695, P1, P12, B128, G27, HPAG1, N6, and J166 (Fig. 4), all of which belong to the *H. pylori* phylogeographic population hpEurope [70]. Thus, the *htrA* sequences confirmed the presence of *H. pylori* in the root canal of the extracted deciduous teeth, and the Neighbour joining tree indicated a phylogeographic population that is matching an origin in a European country.

### Presence of *H. pylori cagA* and typing of EPIYA-motifs

CagA proteins are variable in size, which is mainly attributed to the variable C-terminal repeat regions containing the EPIYA phosphorylation motifs. It was previously reported that *H*. *pylori* worldwide strains can possess one to seven of these EPIYA-sites per CagA protein [71]. Next, we ran specific PCRs with the DNA of the 28 cultured isolates to investigate the presence or absence of the *cagA* gene and for typing of the CagA EPIYA-motifs. The results revealed the presence of 22 CagA-positive and 6 CagA-negative *H. pylori* strains (Table 1). The majority of CagA-positive *H. pylori* contained the 3 EPIYA-sites ABC or 4 EPIYA-sites ABCC (9 strains each). In addition, 4 strains contained 5 EPIYA-sites ABCCC. Thus, the 22 obtained CagA-positive isolates from root canals possessed EPIYA arrangements that are typical for Western-type strains of *H. pylori*.

### Tyrosine phosphorylation of CagA and expression of other *cag*PAI proteins

**Fig. 5:**
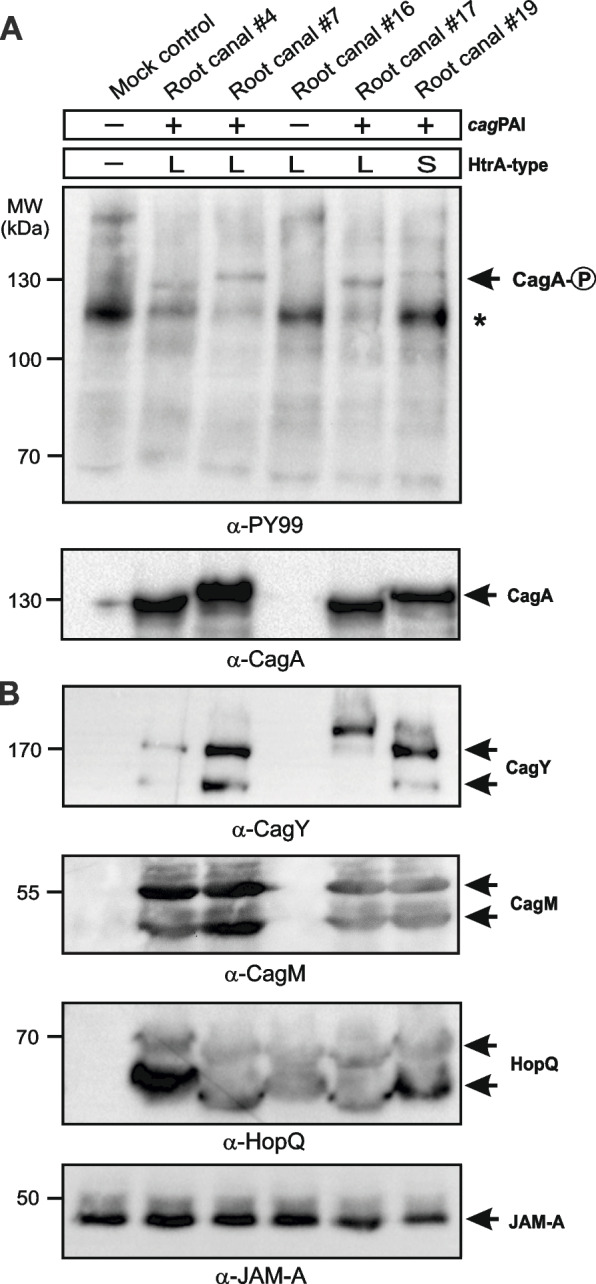
Phosphorylation of CagA in AGS gastric epithelial cells upon infection with representative *H. pylori* isolates from the root canal of teeth. **A** AGS cells were infected for 6 h with the indicated CagA-positive root canal strains (# 4, 7, 17 and 19) and a CagA-negative isolate (# 16) using an MOI of 50. CagA expression and phosphorylation were determined by Western blot analysis using the indicated antibodies. The asterisk marks a phosphorylated 120 kDa host cell protein running below phospho-CagA (arrow). **B** The blots were stripped and reprobed with anti-CagY, anti-CagM and anti-HopQ antibodies. The anti-Jam-A blot served as loading control showing that equal amounts of protein are present in each lane. The results are representative from at least three independent experiments

After delivery into gastric epithelial cells, CagA undergoes phosphorylation at tyrosine residues within the EPIYA-motifs by the oncogenic Src and Abl kinases [18, 72]. In order to study the injection and phosphorylation of CagA, cultured AGS cells were infected with the different *H. pylori* strains for 6 h, and CagA tyrosine phosphorylation was analyzed by Western blotting using anti-CagA antibodies and anti-phosphotyrosine antibodies, respectively (Fig. 5A). The Western blots revealed that all *cagA*-positive strains (as determined by PCR) expressed the CagA protein (Table 1, examples in Fig. 5A). In accordance with the presence of variable EPIYA-motifs, the CagA proteins from the 22 *cagA*-positive strains varied in size from ~ 120 to 140 kDa (Table 1). As expected, all CagAs were phosphorylated by the host cell kinases. In addition, we investigated the expression of T4SS proteins CagY and CagM, which correlated with the expression of CagA (Fig. 5B, Fig. S1). Western blotting further confirmed that the CEACAM receptor binding adhesin HopQ is expressed in all 28 *H. pylori* strains (Fig. 5B, Fig. S1, and Table 1).

### Phase contrast microscopy of *H. pylori-*infected AGS cells

**Fig. 6:**
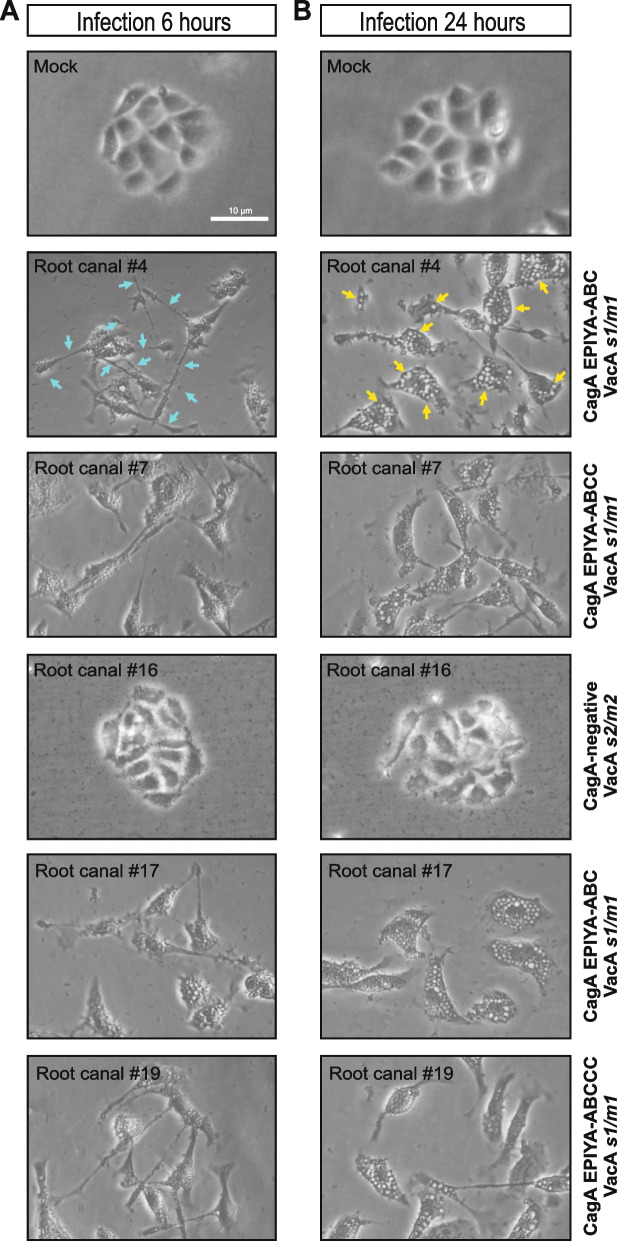
Phase contrast microscopy of AGS cells during infection with representative *H. pylori* isolates from the root canal of teeth. AGS cells were infected for (**A**) 6 h or (**B**) 24 h with the indicated *H. pylori* strains of Fig. 5 using an MOI of 50. The infected cells in the second panels show exemplarily the *H. pylori*-induced elongation phenotype (blue arrows) and vacuole formation (yellow arrows). Note that the cell elongation phenotype and vacuolization are induced in a time-dependent fashion by CagA-positive strains carrying the *s1/m1 vacA* allele (isolates # 4, 7, 17 and 19), but not with the CagA-negative strain carrying the *s2/m2 vacA* allele (isolate # 16). The results are representative from at least three independent experiments

**Fig. 7:**
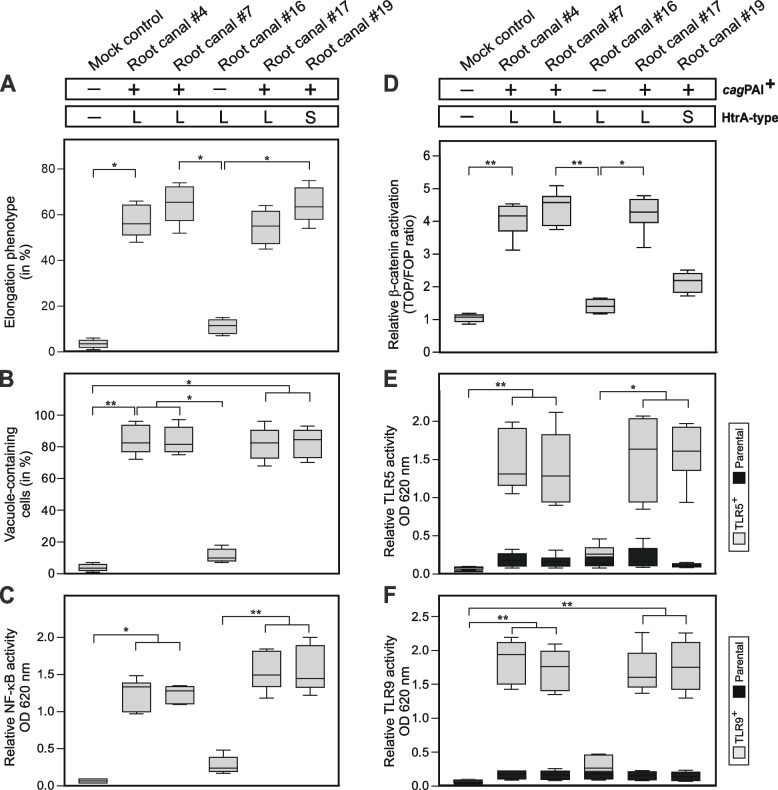
Quantification of cellular phenotypes induced by representative *H. pylori* isolates from the root canal of teeth. AGS cells were infected with the indicated strains of Figs. 5 and 6 using an MOI of 50 for 6 h. AGS cell elongation (**A**), cell vacuolation (**B**), NF-κB activation (**C**) and β-catenin activation (**D**) were quantified. In addition HEK-Blue-TLR5 (**E**), HEK-Blue-TLR9 (**F**) and HEK-Blue-Null1 reporter cells were infected with the same strains for 24 h, and TLR5 and TLR9 activities were quantified. L and S indicate the HtrA variant (171L or 171S) of *H. pylori* isolates. Data are from six independent experiments and represented as box plots, showing the median (line in the box) with the interquartile range. The whiskers indicate the minimum and maximum data values. Statistical significance was defined as *p* ≤ 0.05 ( ∗) and *p* ≤ 0.01 (∗ ∗)

A hallmark of *H. pylori*-infected gastric epithelial cells is the induction of the elongation phenotype by injected CagA and the induction of cellular vacuoles by delivered VacA. To validate the above findings, we next infected AGS cells for 6 h and 24 h, respectively, and monitored whether the 28 isolated *H. pylori* strains induced these typical cellular phenotypes. In agreement with previous studies [73], we detected the pronounced elongation phenotype with all 22 CagA-positive *H. pylori* strains (Fig. 6A, Fig. 7A) that correlated with the appearance of CagA tyrosine phosphorylation (Table 1). In contrast, the elongation phenotype was not seen during infection with CagA-negative isolates (example in Fig. 6A, clone 16). In addition, we monitored the production of VacA-dependent vacuoles in infected AGS cells after 24 h of infection. The ability of *H. pylori* strains to induce cellular vacuoles correlated with the presence of the *s1/m1 vacA* allele, while strains carrying the *s1/m2* or *s2/m2* alleles induced only little or no vacuoles, as expected (Fig. 6B, Fig. 7B, Table 1).

### *H. pylori-*induced NF-κB and β-catenin activation in AGS cells

In *H. pylori*-positive patients, gastric mucosal cytokine and chemokine levels correlate with the intensity of inflammation, and the activation of the pro-inflammatory transcription factor NF-κB is mainly driven by the T4SS-mediated delivery of ADPH [74]. To investigate if the teeth-derived *H. pylori* strains can stimulate the activation of NF-ĸB, we infected AGS cells expressing the NF-ĸB-SEAP reporter for 8 h. Our data show that pronounced NF-ĸB activation was only seen during infection by the 22 *ca*gPAI-positive *H. pylori* isolates carrying a functional T4SS (Fig. 7C, Table 1). In addition, it is described that profound proteolytic cleavage of the adherens junction protein E-cadherin by L-type HtrA expressing *H. pylori* releases β-catenin from the E-cadherin complex [35], followed by CagA-mediated β-catenin delivery into the nucleus and β-catenin/LEF-1-dependent transactivation and cell proliferation [75]. To test if the 28 isolated *H. pylori* strains can stimulate such responses, we utilized TOP-/FOP-flash luciferase reporter assays using polarized AGS cells stably expressing E-cadherin and quantified nuclear β-catenin stimulation upon infection. The data validated that the 8 T4SS-positive L-type *H. pylori*, but not the 20 171S-type bacteria, strongly stimulated β-catenin activity (Fig. 7D, Table 1).

### CagPAI-positive *H. pylori* strains activate TLR5 and TLR9

In order to study if the 28 isolated *H. pylori* strains are able to stimulate the TLR5 receptor [25, 26], HEK293 cells were stably transfected with a plasmid expressing human TLR5 and a NF-κB/AP-1-associated SEAP reporter construct (TLR5^+^), or control cells (Parental) only expressing the NF-κB/AP-1-linked SEAP reporter. The data revealed strong TLR5 activation upon infection with the 22 *cag*PAI-positive *H. pylori*, but not upon infection with the 6 *cag*PAI-negative *H. pylori* strains (Fig. 7E, Table 1). In addition, we infected epithelial HEK293-TLR9 reporter cells with all strains under the same conditions. The results show that the same *cag*PAI-positive isolates, but not *cag*PAI-negative *H. pylori,* activated TLR9 analogous to their capability to activate TLR5 (Fig. 7F, Table 1). Therefore, *H. pylori* isolated from teeth are similar to other strains and activate TLR5 and TLR9 in a T4SS-depending manner.

## Discussion

Even though several studies have previously reported the finding of *H. pylori* DNA in oral samples, a recent review article [38] expressed doubts about the detection of viable *H. pylori* outside the human stomach. The authors questioned the capability to definitively detect live *H. pylori* in the plaque, tongue or saliva by cultivation techniques and claimed that the presence of viable *H. pylori* in the oral cavity remains unclear. This uncertainty prompts the fundamental question of whether *H. pylori* can colonise the oral cavity and, if so, whether colonisation is temporary or permanent and in which oral compartment. In the present study, we investigated the occurrence and characteristics of *H. pylori* in 170 endodontically infected deciduous teeth as compared to plaque. We successfully cultured viable *H. pylori* from the root canal samples of 28 avital deciduous teeth, demonstrating the presence of this bacterium in the oral cavity. Sequencing of the *htrA* genes of the corresponding *H. pylori* isolates revealed new *htrA* haplotypes with up to 98% sequence similarity to *htrA* sequences from *H. pylori* genomes deposited in Genbank (Fig. 4). Some of the new *H. pylori* strains possessed a HtrA with a leucine at amino acid position 171 (171L), a variant that was recently shown to represent a risk genotype for the development of gastric cancer [35]. Furthermore, PCR analyses and Western blotting confirmed the presence and expression of genes that are specific to *H. pylori*, and electron microscopy images visualised characteristic spiral-shaped, *H. pylori*-like organisms adhering to the dentin surface. In addition, we show that during incubation with gastric epithelial cells in vitro, the isolated strains exhibit typical characteristics of an *H. pylori* infection, including host cell vacuolization and elongation as well as activation of host signalling through NF-κB, β-catenin, TLR5 and TLR9 (Table 1). Thus, our study provides clear and irrefutable evidence of the presence of viable *H. pylori* in the root canals of endodontically infected teeth in a large number of patients.

**Fig. 8:**
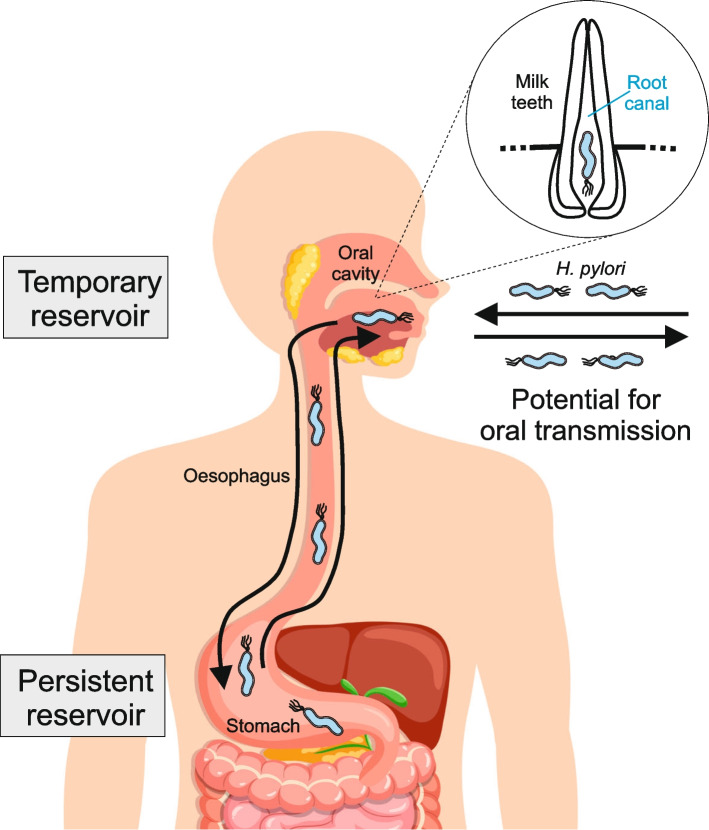
Model for the role of the oral cavity and root canals in deciduous teeth in the transmission and re-infection of the stomach by *H. pylori*. It is proposed that *H. pylori* can be mainly transmitted between human individuals by an oral > oral pathway, e.g. from parents to their children. However, the potential role of the oral cavity as a sporadic or persistent reservoir for *H. pylori* is under debate. We propose that viable *H. pylori* can survive in the protected microaerobic niche in the root canals of deciduous teeth, but not in plaque or saliva. The lack of hygiene in the mouth benefits the survival of viable *H. pylori* in this compartment, which directly influences the duration of oral colonisation and transmission to other persons. Conversely, better oral hygiene or extraction of the corresponding infected teeth can stop the oral colonisation. Thus, we consider the oral environment as a sporadic reservoir for *H. pylori* survival and growth depending on the hygiene. In addition, there is a direct relation between oral and gastric *H. pylori* infection. On one hand, the bacteria can enter the oesophagus via the oral cavity and reach the antrum in the stomach, the preferred habitat of persistent *H. pylori* colonization*.* On the other hand, reflux or vomiting can transfer *H. pylori* from the gastric to the oral compartment, where the bacteria can re-populate the root canals of teeth. Potentially, from the oral cavity bacteria can be transmitted to other persons by the oral-to-oral route. Finally, during anti-*H. pylori* therapy, the bacteria can be eradicated from the stomach, but *H. pylori* in the root canal of teeth are not affected by the therapy. This can explain why sometimes the same bacteria can re-populate the stomach a few weeks after successful antibiotic treatment. Arrows indicate the directions of bacterial transfer. The human thorax picture was designed by brgfx—Freepik.com (https://de.freepik.com/)

In our previous study on a small cohort of three patients we already demonstrated that viable *H. pylori* can be cultured from root canals of deciduous teeth infected with endodontic bacteria, thereby supporting the hypothesis that these root canals may serve as a general reservoir for *H. pylori* [49]. Neither the previous study nor the current study yielded viable *H. pylori* from any of the plaque samples suggesting that the protected area of the root canal may be the most suitable and perhaps sole habitat for *H. pylori* within the oral cavity. However, the electron microscopic pictures revealed that the number of *H. pylori* bacteria is limited in the root canals and that the bacteria share this niche with other microbes, predominantly Gram-positive cocci that compete for the same habitat. It appears that oral hygiene and systemic antibiotic treatment, e.g. eradication measures, are not effective in these areas, as the interior of avital deciduous teeth represents a non-perfused, anaerobic environment. Previous studies proposed that *H. pylori* can be found in plaque, and we were indeed able to amplify *H. pylori*-specific genes from 5 out of 170 plaque samples by PCR. However, stress-induced changes in *H. pylori* makes the cultivation of bacteria from these samples more challenging [40, 76]. In fact, we did not succeed in culturing viable *H. pylori* from any of the 170 plaque samples. Thus, in agreement with previous studies, the plaque on the teeth does probably not represent habitat for viable *H. pylori* [38, 40], in contrast to infected root canals. We therefore propose that *H. pylori*, when present, can survive only for short times in the aerobic environment of saliva and plaque, and that these areas of the oral cavity do not represent a typical reservoir for *H. pylori* survival and growth (Fig. 8).

In this context, it is important to highlight the alarming prevalence of dental caries in children worldwide. According to the Global Burden of Disease Collaborative Network [77], more than 530 million children suffer from caries in the primary dentition, with the majority of cases remaining untreated. This problem is also evident in Germany, where 13.7% of 3-year-olds already have caries, with an average of 3.57 teeth per child affected. Additionally, 50% of 6- to 7-year-olds are affected by tooth decay, and only 26.1% of 3-year-olds and 57.6% of 6- to 7-year-olds receive dental treatment [78]. The high prevalence of endodontic infections and other dental problems indicates that they are widespread and may serve as a reservoir for pathogenic bacteria such as *H. pylori*. Accordingly, a meta-analysis recommended that appropriate professional dental cleaning be performed in conjunction with *H. pylori* eradication therapy to mitigate the risk of subsequent reinfection of the stomach from the oral cavity [79]. Overall, general hygiene deficiencies have been identified as a major risk factor for the development of *H. pylori* infections [80, 81]. It is therefore not surprising that we have found viable *H. pylori* to be commonly present in children with decayed teeth, as caries in the primary dentition is essentially a socially determined hygiene deficiency disease. Obviously, this reservoir for *H. pylori* is removed with the extraction of the avital deciduous teeth. Consequently, rigorous dental treatment and hygiene protocols are essential to mitigate the risk of potential stomach infections that are associated with oral health.

Despite the recent data and the findings from previous research, there are still unanswered questions about the dynamics of *H. pylori* colonisation in the oral cavity and its impact on gastric health. The potential of *H. pylori* to migrate from endodontically infected root canals into the stomach raises questions about the transmission routes of these bacteria. It remains unclear whether these bacteria originate in the stomach and migrate to the root canals, or whether they migrate from the root canals to the stomach. We propose that both pathways are conceivable (Fig. 8). Gastric reflux relocates bacteria from the gastric juice to the oral cavity, where the root canals of avital teeth can serve as a temporary reservoir for *H. pylori*. However, we do not propose that the oral cavity is a permanent reservoir for *H. pylori* because, unlike the situation in the human stomach, the common conditions in the oral environment can change rapidly and *H. pylori* is more under selective pressure by other colonizing and competing microbes. On the other hand, *H. pylori* from the transient niche in decayed teeth can enter the stomach with chewed and swallowed food and saliva as a vessel, and thus, represent a risk factor for a potential re-infection of the stomach after a successful *H. pylori* eradication therapy. Future studies are required to investigate these oral colonization determinants in more detail. By closing these knowledge gaps, we can gain a better understanding of the relationship between oral and gastric infections, which in turn will help to develop more effective prevention and treatment strategies against *H. pylori*. The findings underscore the potential role of teeth as a reservoir for live *H. pylori* and the impact on oral and systemic health.

## Supplementary Information

Supplementary Material 1.

Supplementary Material 2.

Supplementary Material 3.

Supplementary Material 4.

## Data Availability

No datasets were generated or analysed during the current study.

## References

[1] Gonzalez I, Araya P, Schneider I, Lindner C, Rojas A. Pattern recognition receptors and their roles in the host response to *Helicobacter pylori* infection. Future Microbiol. 2021;16:1229–38. 34615380 10.2217/fmb-2021-0106

[2] Wroblewski LE, Peek RM Jr. Clinical pathogenesis, molecular mechanisms of gastric cancer development. Curr Top Microbiol Immunol. 2023;444:25–52. 38231214 10.1007/978-3-031-47331-9_2 PMC10924282

[3] Salama NR, Hartung ML, Muller A. Life in the human stomach: persistence strategies of the bacterial pathogen *Helicobacter pylori*. Nat Rev Microbiol. 2013;11:385–99. 23652324 10.1038/nrmicro3016 PMC3733401

[4] IARC. Schistosomes, liver flukes and *Helicobacter pylori*. IARC Monogr Eval Carcinog Risks Hum. 1994;61:1–241. PMC7681621 7715068

[5] Sung H, Ferlay J, Siegel RL, Laversanne M, Soerjomataram I, Jemal A, Bray F. Global Cancer Statistics 2020: GLOBOCAN Estimates of Incidence and Mortality Worldwide for 36 Cancers in 185 Countries. CA Cancer J Clin. 2021;71:209–49. 33538338 10.3322/caac.21660

[6] Atherton JC, Blaser MJ. Coadaptation of *Helicobacter pylori* and humans: ancient history, modern implications. J Clin Invest. 2009;119:2475–87. 19729845 10.1172/JCI38605 PMC2735910

[7] Kyburz A, Muller A. *Helicobacter pylori* and extragastric diseases. Curr Top Microbiol Immunol. 2017;400:325–47. 28124160 10.1007/978-3-319-50520-6_14

[8] Sachs G, Kraut JA, Wen Y, Feng J, Scott DR. Urea transport in bacteria: acid acclimation by gastric *Helicobacter *spp. J Membr Biol. 2006;212:71–82. 17264989 10.1007/s00232-006-0867-7

[9] Backert S, Haas R, Gerhard M, Naumann M. The *Helicobacter pylori* type IV secretion system encoded by the *cag* pathogenicity island: architecture, function, and signaling. Curr Top Microbiol Immunol. 2017;413:187–220. 29536360 10.1007/978-3-319-75241-9_8

[10] Sheedlo MJ, Ohi MD, Lacy DB, Cover TL. Molecular architecture of bacterial type IV secretion systems. PLoS Pathog. 2022;18:e1010720. 35951533 10.1371/journal.ppat.1010720 PMC9371333

[11] Censini S, Lange C, Xiang Z, Crabtree JE, Ghiara P, Borodovsky M, et al. cag, a pathogenicity island of *Helicobacter pylori*, encodes type I-specific and disease-associated virulence factors. Proc Natl Acad Sci USA. 1996;93:14648–53. 8962108 10.1073/pnas.93.25.14648 PMC26189

[12] Yamaoka Y, Saruuljavkhlan B, Alfaray RI, Linz B. Pathogenomics of *Helicobacter pylori*. Curr Top Microbiol Immunol. 2023;444:117–55. 38231217 10.1007/978-3-031-47331-9_5

[13] Fischer W, Tegtmeyer N, Stingl K, Backert S. Four chromosomal type IV secretion systems in *Helicobacter pylori*: composition structure and function. Front Microbiol. 2020;11:1592. 32754140 10.3389/fmicb.2020.01592 PMC7366825

[14] Moonens K, Hamway Y, Neddermann M, Reschke M, Tegtmeyer N, Kruse T, et al. *Helicobacter pylori *adhesin HopQ disrupts trans dimerization in human CEACAMs. EMBO J. 2018;37:e98665. 29858229 10.15252/embj.201798665 PMC6028033

[15] Tegtmeyer N, Harrer A, Schmitt V, Singer BB, Backert S. Expression of CEACAM1 or CEACAM5 in AZ-521 cells restores the type IV secretion deficiency for translocation of CagA by *Helicobacter pylori*. Cell Microbiol. 2019;21:e12965. 30321907 10.1111/cmi.12965

[16] Backert S, Ziska E, Brinkmann V, Zimny-Arndt U, Fauconnier A, Jungblut PR, Naumann M, Meyer TF. Translocation of the *Helicobacter pylori* CagA protein in gastric epithelial cells by a type IV secretion apparatus. Cell Microbiol. 2000;2:155–64. 11207572 10.1046/j.1462-5822.2000.00043.x

[17] Mueller D, Tegtmeyer N, Brandt S, Yamaoka Y, De Poire E, Sgouras D, et al. c-Src and c-Abl kinases control hierarchic phosphorylation and function of the CagA effector protein in Western and East Asian *Helicobacter pylori *strains. J Clin Invest. 2012;122:1553–66. 22378042 10.1172/JCI61143 PMC3314471

[18] Selbach M, Moese S, Hauck CR, Meyer TF, Backert S. Src is the kinase of the *Helicobacter pylori* CagA protein in vitro and in vivo. J Biol Chem. 2002;277:6775–8. 11788577 10.1074/jbc.C100754200

[19] Hatakeyama M. Impact of the *Helicobacter pylori *Oncoprotein CagA in Gastric Carcinogenesis. Curr Top Microbiol Immunol. 2023;444:239–57. 38231221 10.1007/978-3-031-47331-9_9

[20] Tegtmeyer N, Neddermann M, Asche CI, Backert S. Subversion of host kinases: a key network in cellular signaling hijacked by *Helicobacter pylori *CagA. Mol Microbiol. 2017;105:358–72. 28508421 10.1111/mmi.13707

[21] Faass L, Stein SC, Hauke M, Gapp M, Albanese M, Josenhans C. Contribution of heptose metabolites and the *cag *pathogenicity island to the activation of monocytes/macrophages by *Helicobacter pylori.* Front Immunol. 2021;12:632154. 34093525 10.3389/fimmu.2021.632154 PMC8174060

[22] Gall A, Gaudet RG, Gray-Owen SD, Salama NR. TIFA signaling in gastric epithelial cells initiates the cag type 4 secretion system-dependent innate immune response to *Helicobacter pylori* infection. mBio. 2017;8:e01168–17. 28811347 10.1128/mBio.01168-17 PMC5559637

[23] Maubach G, Lim MCC, Sokolova O, Backert S, Meyer TF, Naumann M. TIFA has dual functions in *Helicobacter pylori*-induced classical and alternative NF-kappaB pathways. EMBO Rep. 2021;22:e52878. 34328245 10.15252/embr.202152878 PMC8419686

[24] Pfannkuch L, Hurwitz R, Traulsen J, Sigulla J, Poeschke M, Matzner L, et al. ADP heptose, a novel pathogen-associated molecular pattern identified in *Helicobacter pylori*. FASEB J. 2019;33:9087–99. 31075211 10.1096/fj.201802555R PMC6662969

[25] Pachathundikandi SK, Tegtmeyer N, Arnold IC, Lind J, Neddermann M, Falkeis-Veits C, et al. T4SS-dependent TLR5 activation by *Helicobacter pylori *infection. Nat Commun. 2019;10:5717. 31844047 10.1038/s41467-019-13506-6 PMC6915727

[26] Tegtmeyer N, Neddermann M, Lind J, Pachathundikandi SK, Sharafutdinov I, Gutierrez-Escobar AJ, et al. Toll-like Receptor 5 Activation by the CagY Repeat Domains of *Helicobacter pylori*. Cell Rep. 2020;32:108159. 32937132 10.1016/j.celrep.2020.108159

[27] Lin AS, Dooyema SDR, Frick-Cheng AE, Harvey ML, Suarez G, Loh JT, et al. Bacterial energetic requirements for *Helicobacter pylori* Cag type IV secretion system-dependent alterations in gastric epithelial cells. Infect Immun. 2020;88:e00790–e819. 31712269 10.1128/IAI.00790-19 PMC6977121

[28] Tegtmeyer N, Linz B, Yamaoka Y, Backert S. Unique TLR9 Activation by *Helicobacter pylori* Depends on the cag T4SS, But Not on VirD2 Relaxases or VirD4 Coupling Proteins. Curr Microbiol. 2022;79:121. 35239059 10.1007/s00284-022-02813-9 PMC8894178

[29] Varga MG, Shaffer CL, Sierra JC, Suarez G, Piazuelo MB, Whitaker ME, et al. Pathogenic *Helicobacter pylori* strains translocate DNA and activate TLR9 via the cancer-associated cag type IV secretion system. Oncogene. 2016;35:6262–9. 27157617 10.1038/onc.2016.158 PMC5102820

[30] Varga MG, Piazuelo MB, Romero-Gallo J, Delgado AG, Suarez G, Whitaker ME, et al. TLR9 activation suppresses inflammation in response to *Helicobacter pylori *infection. Am J Physiol Gastrointest Liver Physiol. 2016;311:G852–8. 27758771 10.1152/ajpgi.00175.2016 PMC5130555

[31] Foegeding NJ, Caston RR, McClain MS, Ohi MD, Cover TL. An Overview of *Helicobacter pylori* VacA Toxin Biology. Toxins (Basel). 2016;8:173. 27271669 10.3390/toxins8060173 PMC4926140

[32] Sharafutdinov I, Knorr J, SoltanEsmaeili D, Backert S, Tegtmeyer N. Cortactin Promotes Effective AGS Cell Scattering by *Helicobacter pylori* CagA, but Not Cellular Vacuolization and Apoptosis Induced by the Vacuolating Cytotoxin VacA. Pathogens. 2021;11:3. 35055951 10.3390/pathogens11010003 PMC8777890

[33] Linz B, Sticht H, Tegtmeyer N, Backert S. Cancer-associated SNPs in bacteria: lessons from *Helicobacter pylori*. Trends Microbiol. 2024;32:847–57. 10.1016/j.tim.2024.02.001 38485609

[34] Schmidt TP, Perna AM, Fugmann T, Bohm M, Hiss J, Haller S, et al. Identification of E-cadherin signature motifs functioning as cleavage sites for *Helicobacter pylori* HtrA. Sci Rep. 2016;6:23264. 26983597 10.1038/srep23264 PMC4794652

[35] Sharafutdinov I, Tegtmeyer N, Linz B, Rohde M, Vieth M, Tay AC-Y, et al. A single nucleotide polymorphism in *Helicobacter pylori *promotes gastric cancer development. Cell Host Microbe. 2023;31:1345–58. 10.1016/j.chom.2023.06.016 37490912

[36] Kayali S, Manfredi M, Gaiani F, Bianchi L, Bizzarri B, Leandro G, Di Mario F, De’ Angelis GL. *Helicobacter pylori*, transmission routes and recurrence of infection: state of the art. Acta Biomed. 2018;89:72–6. 30561421 10.23750/abm.v89i8-S.7947 PMC6502203

[37] Tsimpiris A, Tsolianos I, Grigoriadis A, Moschos I, Goulis DG, Kouklakis G. Association of chronic periodontitis with *Helicobacter pylori* infection in stomach or mouth: a systematic review and meta-analysis. Eur J Dent. 2023;17:270–82. 36400109 10.1055/s-0042-1756690 PMC10329527

[38] Mao X, Jakubovics NS, Bachle M, Buchalla W, Hiller KA, Maisch T, et al. Colonization of *Helicobacter pylori* in the oral cavity - an endless controversy? Crit Rev Microbiol. 2021;47:612–29. 10.1080/1040841X.2021.1907740. 33899666 10.1080/1040841X.2021.1907740

[39] Parsonnet J, Shmuely H, Haggerty T. Fecal and oral shedding of *Helicobacter pylori* from healthy infected adults. JAMA. 1999;282:2240–5. 10605976 10.1001/jama.282.23.2240

[40] Zhang L, Chen X, Ren B, Zhou X, Cheng L. *Helicobacter pylori* in the Oral Cavity: Current Evidence and Potential Survival Strategies. Int J Mol Sci. 2022;23:13646. 10.3390/ijms232113646 PMC9657019 36362445

[41] Burgers R, Schneider-Brachert W, Reischl U, Behr A, Hiller KA, Lehn N, et al. *Helicobacter pylori* in human oral cavity and stomach. Eur J Oral Sci. 2008;116:297–304. 18705796 10.1111/j.1600-0722.2008.00543.x

[42] Dane A, Gurbuz T. Clinical comparative study of the effects of *Helicobacter pylori *colonization on oral health in children. Pak J Med Sci. 2016;32:969–73. 27648050 10.12669/pjms.324.10034 PMC5017113

[43] Hamada M, Nomura R, Matayoshi S, Ogaya Y, Kameyama H, Uzawa N, Nakano K. Detection of Helicobacter pylori from Extracted Teeth of a Patient with Idiopathic Thrombocytopenic Purpura. Microorganisms. 2022;10:2285. 10.3390/microorganisms10112285 PMC9693563 36422356

[44] Krajden S, Fuksa M, Anderson J, Kempston J, Boccia A, Petrea C, et al. Examination of human stomach biopsies, saliva, and dental plaque for *Campylobacter pylori*. J Clin Microbiol. 1989;27:1397–8. 2754008 10.1128/jcm.27.6.1397-1398.1989 PMC267568

[45] Nagata R, Sato H, Takenaka S, Yokoyama J, Terai S, Mimuro H, Noiri Y. Analysis of Genetic Relatedness between Gastric and Oral *Helicobacter pylori* in Patients with Early Gastric Cancer Using Multilocus Sequence Typing. Int J Mol Sci. 2023;24:2211. 10.3390/ijms24032211 PMC9917182 36768541

[46] Nguyen AM, Engstrand L, Genta RM, Graham DY, El-Zaatari FA. Detection of *Helicobacter pylori* in dental plaque by reverse transcription-polymerase chain reaction. J Clin Microbiol. 1993;31:783–7. 8463387 10.1128/jcm.31.4.783-787.1993 PMC263562

[47] Nguyen AM, el-Zaatari FA, Graham DY. *Helicobacter pylori *in the oral cavity. A critical review of the literature. Oral Surg Oral Med Oral Pathol Oral Radiol Endod. 1995;79:705–9. 10.1016/s1079-2104(05)80304-x 7621027

[48] Sruthi MA, Mani G, Ramakrishnan M, Selvaraj J. Dental caries as a source of *Helicobacter pylor*i infection in children: An RT-PCR study. Int J Paediatr Dent. 2023;33:82–8. 35771167 10.1111/ipd.13017

[49] Hirsch C, Tegtmeyer N, Rohde M, Rowland M, Oyarzabal OA, Backert S. Live *Helicobacter pylori* in the root canal of endodontic-infected deciduous teeth. J Gastroenterol. 2012;47:936–40. 22722905 10.1007/s00535-012-0618-8

[50] Tomb JF, White O, Kerlavage AR, Clayton RA, Sutton GG, Fleischmann RD, et al. The complete genome sequence of the gastric pathogen *Helicobacter pylori.* Nature. 1997;388:539–47. 9252185 10.1038/41483

[51] Schoep TD, Fulurija A, Good F, Lu W, Himbeck RP, Schwan C, et al. Surface properties of *Helicobacter pylori* urease complex are essential for persistence. PLoS ONE. 2010;5:e15042. 21124783 10.1371/journal.pone.0015042 PMC2993952

[52] Oliveira MJ, Costa AM, Costa AC, Ferreira RM, Sampaio P, Machado JC, et al. CagA associates with c-Met, E-cadherin, and p120-catenin in a multiproteic complex that suppresses *Helicobacter pylori*-induced cell-invasive phenotype. J Infect Dis. 2009;200:745–55. 19604117 10.1086/604727

[53] Tegtmeyer N, Wessler S, Necchi V, Rohde M, Harrer A, Rau TT, et al. *Helicobacter pylori *Employs a Unique Basolateral Type IV Secretion Mechanism for CagA Delivery. Cell Host Microbe. 2017;22:552–560.e5. 29024645 10.1016/j.chom.2017.09.005

[54] Tegtmeyer N, SoltanEsmaeili D, Sharafutdinov I, Knorr J, Naumann M, Alter T, Backert S. Importance of cortactin for efficient epithelial NF-κB activation by *Helicobacter pylori*, *Salmonella enterica* and *Pseudomonas aeruginosa*, but not *Campylobacter* spp. Eur J Microbiol Immunol. 2022;11:95–103. 10.1556/1886.2021.00023 PMC8830411 35060920

[55] Andersen-Nissen E, Smith KD, Strobe KL, Barrett SL, Cookson BT, Logan SM, Aderem A. Evasion of Toll-like receptor 5 by flagellated bacteria. Proc Natl Acad Sci USA. 2005;102:9247–52. 15956202 10.1073/pnas.0502040102 PMC1166605

[56] Tegtmeyer N, Rivas Traverso F, Rohde M, Oyarzabal OA, Lehn N, Schneider-Brachert W, Ferrero RL, Fox JG, Berg DE, Backert S. Electron microscopic, genetic and protein expression analyses of *Helicobacter acinonychis* strains from a Bengal tiger. PLoS ONE. 2013;8:e71220. 23940723 10.1371/journal.pone.0071220 PMC3733902

[57] Sundrud MS, Torres VJ, Unutmaz D, Cover TL. Inhibition of primary human T cell proliferation by *Helicobacter pylori *vacuolating toxin (VacA) is independent of VacA effects on IL-2 secretion. Proc Natl Acad Sci USA. 2004;101:7727–32. 15128946 10.1073/pnas.0401528101 PMC419674

[58] Tegtmeyer N, Zabler D, Schmidt D, Hartig R, Brandt S, Backert S. Importance of EGF receptor, HER2/Neu and Erk1/2 kinase signalling for host cell elongation and scattering induced by the *Helicobacter pylori* CagA protein: antagonistic effects of the vacuolating cytotoxin VacA. Cell Microbiol. 2009;11:488–505. 19046339 10.1111/j.1462-5822.2008.01269.x

[59] Albrecht N, Tegtmeyer N, Sticht H, Skorko-Glonek J, Backert S. Amino-terminal processing of *Helicobacter pylori* serine protease HtrA: role in oligomerization and activity regulation. Front Microbiol. 2018;9:642. 29713313 10.3389/fmicb.2018.00642 PMC5911493

[60] Kwok T, Zabler D, Urman S, Rohde M, Hartig R, Wessler S, Misselwitz R, Berger J, Sewald N, König W, Backert S. *Helicobacter *exploits integrin for type IV secretion and kinase activation. Nature. 2007;449:862–6. 10.1038/nature06187. 17943123 10.1038/nature06187

[61] Moese S, Selbach M, Zimny-Arndt U, Jungblut PR, Meyer TF, Backert S. Identification of a tyrosine-phosphorylated 35 kDa carboxy-terminal fragment (p35CagA) of the *Helicobacter pylori* CagA protein in phagocytic cells: processing or breakage? Proteomics. 1:618–29. 10.1002/1615-9861(200104)1:4<618::AID-PROT618>3.0.CO;2-C. 10.1002/1615-9861(200104)1:4<618::AID-PROT618>3.0.CO;2-C 11681214

[62] Harrer A, Bücker R, Boehm M, Zarzecka U, Tegtmeyer N, Sticht H, Schulzke JD, Backert S. *Campylobacter jejuni *enters gut epithelial cells and impairs intestinal barrier function through cleavage of occludin by serine protease HtrA. Gut Pathog. 2019;4:2019. 10.1186/s13099-019-0283-z.eCollection. 10.1186/s13099-019-0283-z PMC6373145 30805031

[63] Argent RH, Zhang Y, Atherton JC. Simple method for determination of the number of *Helicobacter pylori* CagA variable-region EPIYA tyrosine phosphorylation motifs by PCR. J Clin Microbiol. 2005;43:791–5. 10.1128/JCM.43.2.791-795.2005 PMC548073 15695681

[64] van Doorn LJ, Figueiredo C, Sanna R, Pena S, Midolo P, Ng EK, Atherton JC, et al. Expanding allelic diversity of* Helicobacter pylori *vacA. J Clin Microbiol. 1998;36:2597–603. 9705399 10.1128/jcm.36.9.2597-2603.1998 PMC105169

[65] Kumar S, Stecher G, Li M, Knyaz C, Tamura K. MEGA X: molecular evolutionary genetics analysis across computing platforms. Mol Biol Evol. 2018;35:1547–9. 29722887 10.1093/molbev/msy096 PMC5967553

[66] Harper CG, Feng Y, Xu S, Taylor NS, Kinsel M, Dewhirst FE, et al. *Helicobacter cetorum *sp. nov., a urease-positive *Helicobacter* species isolated from dolphins and whales. J Clin Microbiol. 2002;40:4536–43. 12454148 10.1128/JCM.40.12.4536-4543.2002 PMC154630

[67] Kersulyte D, Rossi M, Berg DE. Sequence divergence and conservation in genomes of *Helicobacter cetorum *strains from a dolphin and a whale. PLoS ONE. 2013;8:e83177. 24358262 10.1371/journal.pone.0083177 PMC3866246

[68] Eppinger M, Baar C, Linz B, Raddatz G, Lanz C, Keller H, et al. Who ate whom? Adaptive *Helicobacter* genomic changes that accompanied a host jump from early humans to large felines. PLoS Genet. 2006;2:e120. 16789826 10.1371/journal.pgen.0020120 PMC1523251

[69] Bansil R, Constantino MA, Su-Arcaro C, Liao W, Shen Z, Fox JG. Motility of Different Gastric *Helicobacter *spp. Microorganisms. 2023;11:634. 10.3390/microorganisms11030634 PMC10058440 36985208

[70] Linz B, Balloux F, Moodley Y, Manica A, Liu H, Roumagnac P, et al. An African origin for the intimate association between humans and *Helicobacter pylori*. Nature. 2007;445:915–8. 17287725 10.1038/nature05562 PMC1847463

[71] Backert S, Tegtmeyer N, Selbach M. The versatility of *Helicobacter pylori* CagA effector protein functions: the master key hypothesis. Helicobacter. 2010;15:163–76. 20557357 10.1111/j.1523-5378.2010.00759.x

[72] Tammer I, Brandt S, Hartig R, Konig W, Backert S. Constitutive activation of c-Abl tyrosine kinase and the induction of cell scattering by *Helicobacter pylori*. Int J Med Microbiol. 2006;296:172.

[73] Backert S, Moese S, Selbach M, Brinkmann V, Meyer TF. Phosphorylation of tyrosine 972 of the *Helicobacter pylori* CagA protein is essential for induction of a scattering phenotype in gastric epithelial cells. Mol Microbiol. 2001;42:631–44. 11722731 10.1046/j.1365-2958.2001.02649.x

[74] Naumann M, Ferino L, Sharafutdinov I, Backert S. Gastric Epithelial barrier disruption, inflammation and oncogenic signal transduction by *Helicobacter pylori*. Curr Top Microbiol Immunol. 2023;444:207–38. 38231220 10.1007/978-3-031-47331-9_8

[75] Franco AT, Israel DA, Washington MK, Krishna U, Fox JG, Rogers AB, et al. Activation of beta-catenin by carcinogenic *Helicobacter pylori*. Proc Natl Acad Sci USA. 2005;102:10646–51. 16027366 10.1073/pnas.0504927102 PMC1180811

[76] Azevedo NF, Almeida C, Cerqueira L, Dias S, Keevil CW, Vieira MJ. Coccoid form of *Helicobacter pylori* as a morphological manifestation of cell adaptation to the environment. Appl Environ Microbiol. 2007;73:3423–7. 17400788 10.1128/AEM.00047-07 PMC1907093

[77] GBD. Global, regional, and national incidence, prevalence, and years lived with disability for 354 diseases and injuries for 195 countries and territories, 1990–2017: a systematic analysis for the Global Burden of Disease Study 2017. Lancet. 2018;392:1789–1858. 10.1016/S0140-6736(18)32279-7 PMC6227754 30496104

[78] Basner R, Santamaria RM, Schmoeckel J, Schüler E, Splieth C (2017) Epidemiologische Begleituntersuchung zur Gruppenprophylaxe 2016. Deutsche Arbeitsgemeinschaft für Jugendzahnpflege e.V. (DAJ). https://www.daj.de/fileadmin/user_upload/PDF_ Downloads/Epi_2016/Epi_final_BB1801_final.pdf.

[79] Zou QH, Li RQ. *Helicobacter pylori* in the oral cavity and gastric mucosa: a meta-analysis. J Oral Pathol Med. 2011;40:317–24. 21294774 10.1111/j.1600-0714.2011.01006.x

[80] Lee YY, Mahendra Raj S, Graham DY. *Helicobacter pylori *infection–a boon or a bane: lessons from studies in a low-prevalence population. Helicobacter. 2013;18:338–46. 23607896 10.1111/hel.12058 PMC3974589

[81] Onosakponome EO, Adedokun AA, Wogu MN. Assessment of Risk Factors and Outcome of Co-Infection of Soil Transmitted Helminths and *H. pylori* among School Age Children Living in Riverine Slum Settlements in Port Harcourt, Nigeria. Int J Trop Dis & Health. 2022;43:12–17.

