# Supplementary figures and images for: Cultivation and molecular characterization of viable Helicobacter pylori from the root canal of 170 deciduous teeth of children
Source: Cell Commun Signal. 2024 Dec 3;22:578. doi: 10.1186/s12964-024-01948-5 (PMC11613870; doi:10.1186/s12964-024-01948-5)

Figure S1

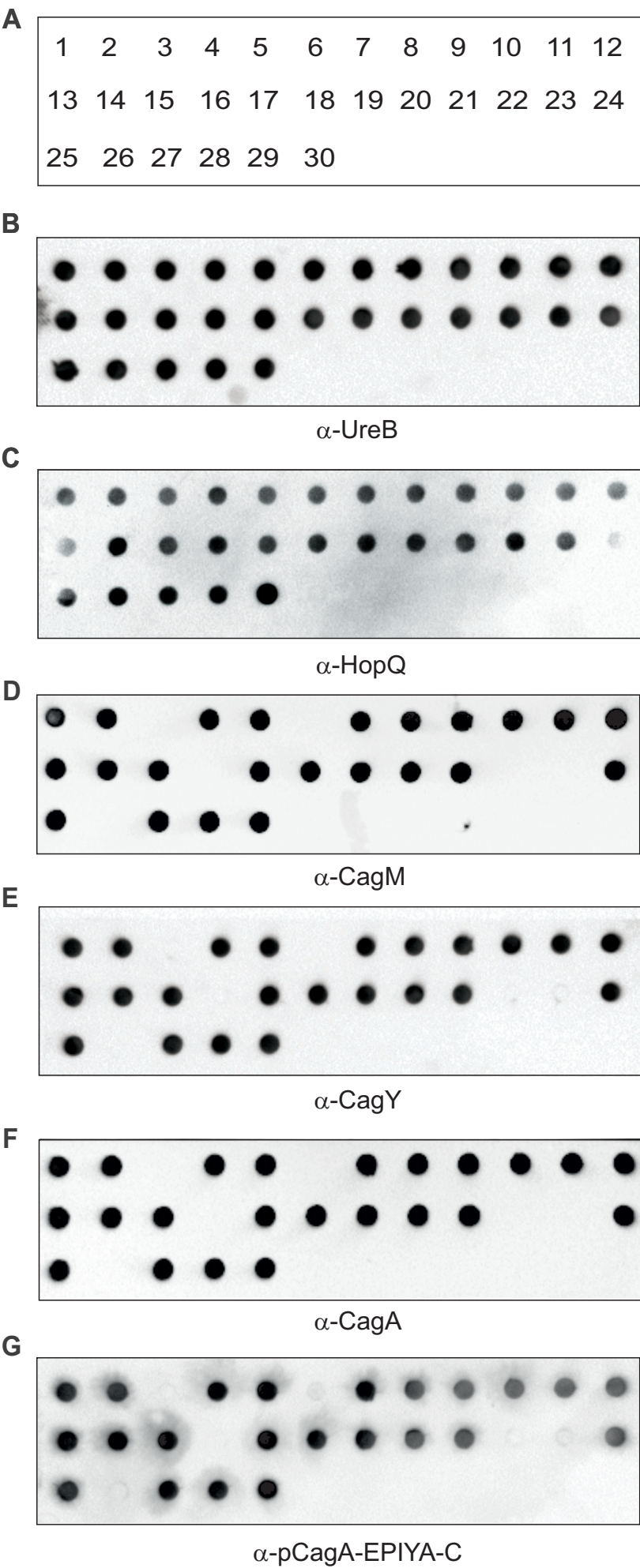

Supplement: Supplementary file 1 — Supplementary Material 1. [file 12964_2024_1948_MOESM1_ESM.pdf]
