# Supplementary material for: Cultivation and molecular characterization of viable Helicobacter pylori from the root canal of 170 deciduous teeth of children
Source: Cell Commun Signal. 2024 Dec 3;22:578. doi: 10.1186/s12964-024-01948-5 (PMC11613870; doi:10.1186/s12964-024-01948-5)
Supplement: Supplementary file 2 — Supplementary Material 2. [file 12964_2024_1948_MOESM2_ESM.pdf]

Table S1 (extended for Reviewer-1) *H. pylori*-positive Patient Demographics and Tooth Extraction Details by Type and Region

Strain # neu	Age	Sex	Number of extracted teeth (infected/total)	Infected tooth region*	Type of uninfected teeth*
SBA-01	3	m	1/6	Molar (upper jaw)	Molar, Incisors (upper jaw)
SBA-02	9	f	1/2	Molar (64)	Molar (74)
SBA-03	5	m	1/4	Molar (54)	Molars (75, 74, 84)
SBA-04	6	f	1/4	Molar (54)	Molars (55, 65, 74)
SBA-05	9	m	1/2	Molar (65)	Molar (84)
SBA-06	3	f	2/6	Molars (74, 54)	Incisors (62, 61, 51, 52)
SBA-07	8	m	1/2	Molar (54)	Molar (84)
SBA-08	6	f	2/9	Molars (upper jaw)	Molar, Canines (upper and lower jaw)
SBA-09	2	f	1/6	Molar (upper jaw)	Molars, Incisors (upper jaw)
SBA-10	8	m	1/3	Molar (upper jaw)	Molars (upper and lower jaw)
SBA-11	5	m	1/7	Incisor (upper jaw)	Molars, Incisors (upper and lower jaw)
SBA-12	5	f	1/3	Molar (64)	Molars (74, 84)
SBA-13	7	m	1/2	Molar (85)	Incisor (62)
SBA-14	7	m	1/1	Molar (54)	
SBA-15	7	m	1/7	Molar	Molars (upper and lower jaw), Canine, Incisors (upper jaw)
SBA-16	2	f	1/8	Molar (upper jaw)	Molars (upper and lower jaw), Incisors (upper jaw)
SBA-17	5	m	2/12	Molar, Canine (upper jaw)	Molars, Canines, Incisors (upper and lower jaw)
SBA-18	5	m	2/12	Molar, Canine (upper jaw)	Molars, Canines, Incisors (upper and lower jaw)
SBA-19	6	f	2/9	Molars (upper jaw)	Molar, Canines (upper and lower jaw)
SBA-20	4	m	1/6	Molar (upper jaw)	Molars (upper and lower jaw), Incisors (upper jaw)
SBA-21	6	m	1/3	Molar (64)	Molars (84, 85)
SBA-22	11	m	1/2	Molar (55)	Molar (54)
SBA-23	3	f	2/7	Molars (64, 65)	Canine, Incisors (upper jaw)
SBA-24	3	f	2/7	Molars (64, 65)	Canine, Incisors (upper jaw)
SBA-25	5	m	1/4	Molar (55)	Molars (54, 85, 84)
SBA-26	3	f	2/6	Molars (74, 54)	Incisors (62, 61, 51, 52)
SBA-27	6	m	1/7	Molar (upper jaw)	Molars (upper and lower jaw), Canine (73, 83)
SBA-28	5	f	1/6	Incisor (62)	Molars (upper and lower jaw)

* Lists the types of infected and uninfected teeth, with tooth designations in parentheses where available, according to the international tooth numbering system. Includes molars, incisors, and canines, specified by their jaw position (upper or lower).
