# Supplementary material for: Cultivation and molecular characterization of viable Helicobacter pylori from the root canal of 170 deciduous teeth of children
Source: Cell Commun Signal. 2024 Dec 3;22:578. doi: 10.1186/s12964-024-01948-5 (PMC11613870; doi:10.1186/s12964-024-01948-5)
Supplement: Supplementary file 3 — Supplementary Material 3. [file 12964_2024_1948_MOESM3_ESM.pdf]

Supplementary Table S1: Demographics and extraction details of *H. pylori*-positive tooth by oral region.

Strain #	Age	Sex	Number of extracted teeth (infected/total)	Infected tooth region*	Uninfected tooth region*
SBA-01	3	m	1/6	Posterior	Posterior, Anterior
SBA-02	9	f	1/2	Posterior	Posterior
SBA-03	5	m	1/4	Posterior	Posterior
SBA-04	6	f	1/4	Posterior	Posterior
SBA-05	9	m	1/2	Posterior	Posterior
SBA-06	3	f	2/6	Posterior	Anterior
SBA-07	8	m	1/2	Posterior	Posterior
SBA-08	6	f	2/9	Posterior	Posterior, Anterior
SBA-09	2	f	1/6	Posterior	Posterior, Anterior
SBA-10	8	m	1/3	Posterior	Posterior
SBA-11	5	m	1/7	Anterior	Posterior, Anterior
SBA-12	5	f	1/3	Posterior	Posterior
SBA-13	7	m	1/2	Posterior	Anterior
SBA-14	7	m	1/1	Posterior	Posterior, Anterior
SBA-15	7	m	1/7	Posterior	Posterior , Anterior
SBA-16	2	f	1/8	Posterior	Posterior, Anterior
SBA-17	5	m	2/12	Posterior, Anterior	Posterior, Anterior
SBA-18	5	m	2/12	Posterior, Anterior	Posterior, Anterior
SBA-19	6	f	2/9	Posterior	Posterior, Anterior
SBA-20	4	m	1/6	Posterior	Posterior, Anterior
SBA-21	6	m	1/3	Posterior	Posterior
SBA-22	11	m	1/2	Posterior	Posterior
SBA-23	3	f	2/7	Posterior	Anterior
SBA-24	3	f	2/7	Posterior	Anterior
SBA-25	5	m	1/4	Posterior	Posterior
SBA-26	3	f	2/6	Posterior	Anterior
SBA-27	6	m	1/7	Posterior	Posterior, Anterior
SBA-28	5	f	1/6	Anterior	Posterior

* These columns Indicate whether the infected or uninfected teeth are in the anterior (front teeth: incisors and canines) or posterior (back teeth: molars) regions of the oral cavity.
