# Supplementary material for: Cultivation and molecular characterization of viable Helicobacter pylori from the root canal of 170 deciduous teeth of children
Source: Cell Commun Signal. 2024 Dec 3;22:578. doi: 10.1186/s12964-024-01948-5 (PMC11613870; doi:10.1186/s12964-024-01948-5)
Supplement: Supplementary file 4 — Supplementary Material 4. [file 12964_2024_1948_MOESM4_ESM.pdf]

Figure 3

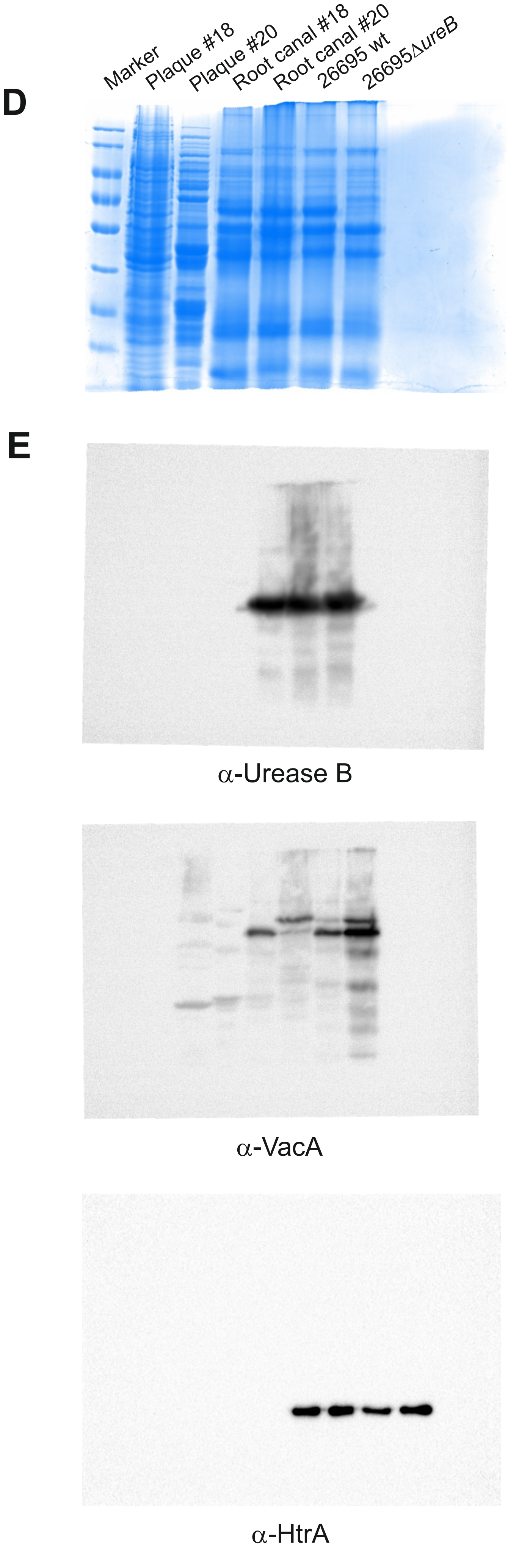

D
Marker
Plaque #18
Plaque #20
Root canal #18
Root canal #20
26695 wt
26695Δ*ureB*
E
α-Urease B
α-VacA
α-HtrA

Figure 5

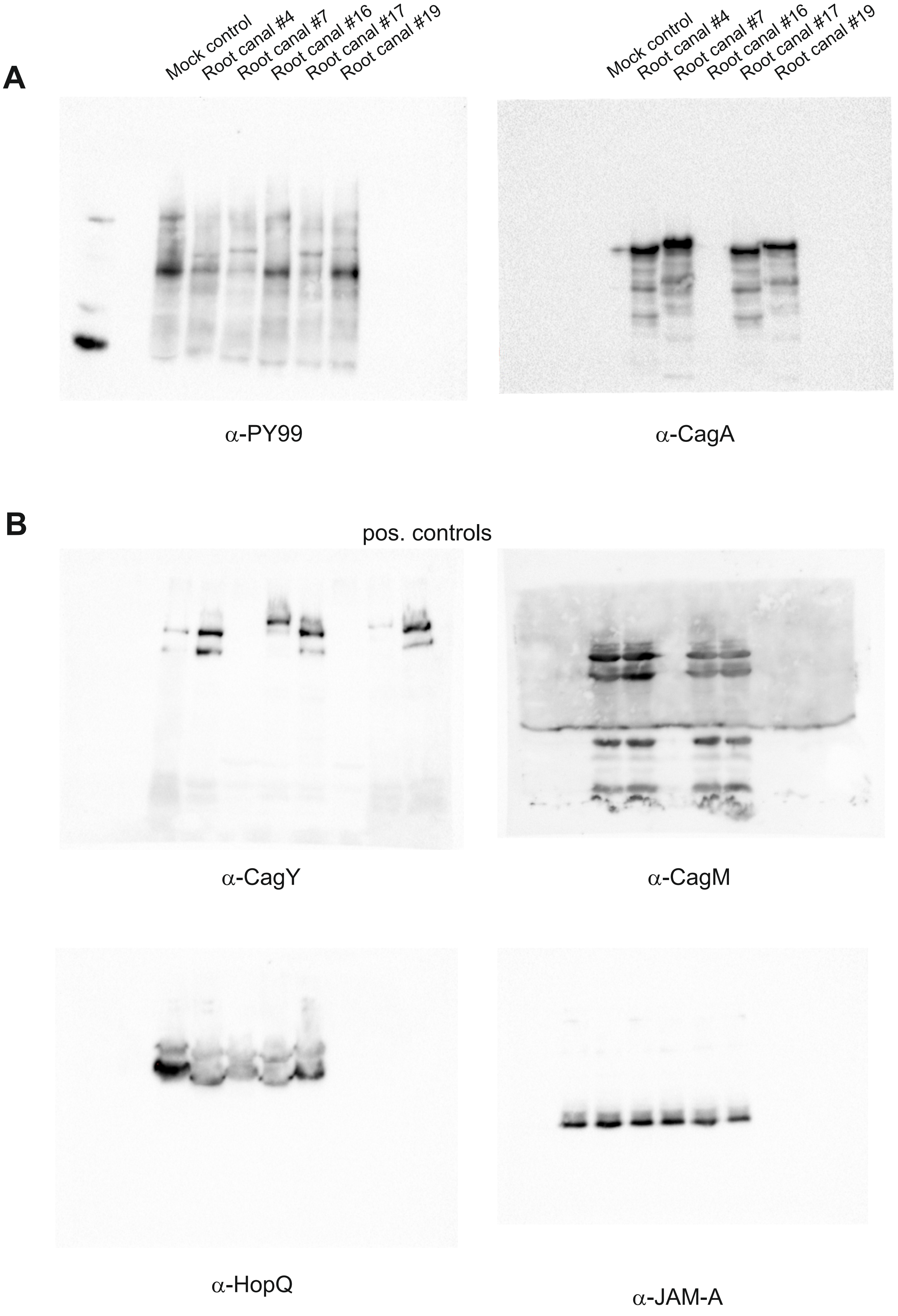
A
Mock control
Root canal #4
Root canal #7
Root canal #16
Root canal #17
Root canal #19
α-PY99
Mock control
Root canal #4
Root canal #7
Root canal #16
Root canal #17
Root canal #19
α-CagA
B
pos. controls
α-CagY
α-CagM
α-HopQ
α-JAM-A

Figure S1

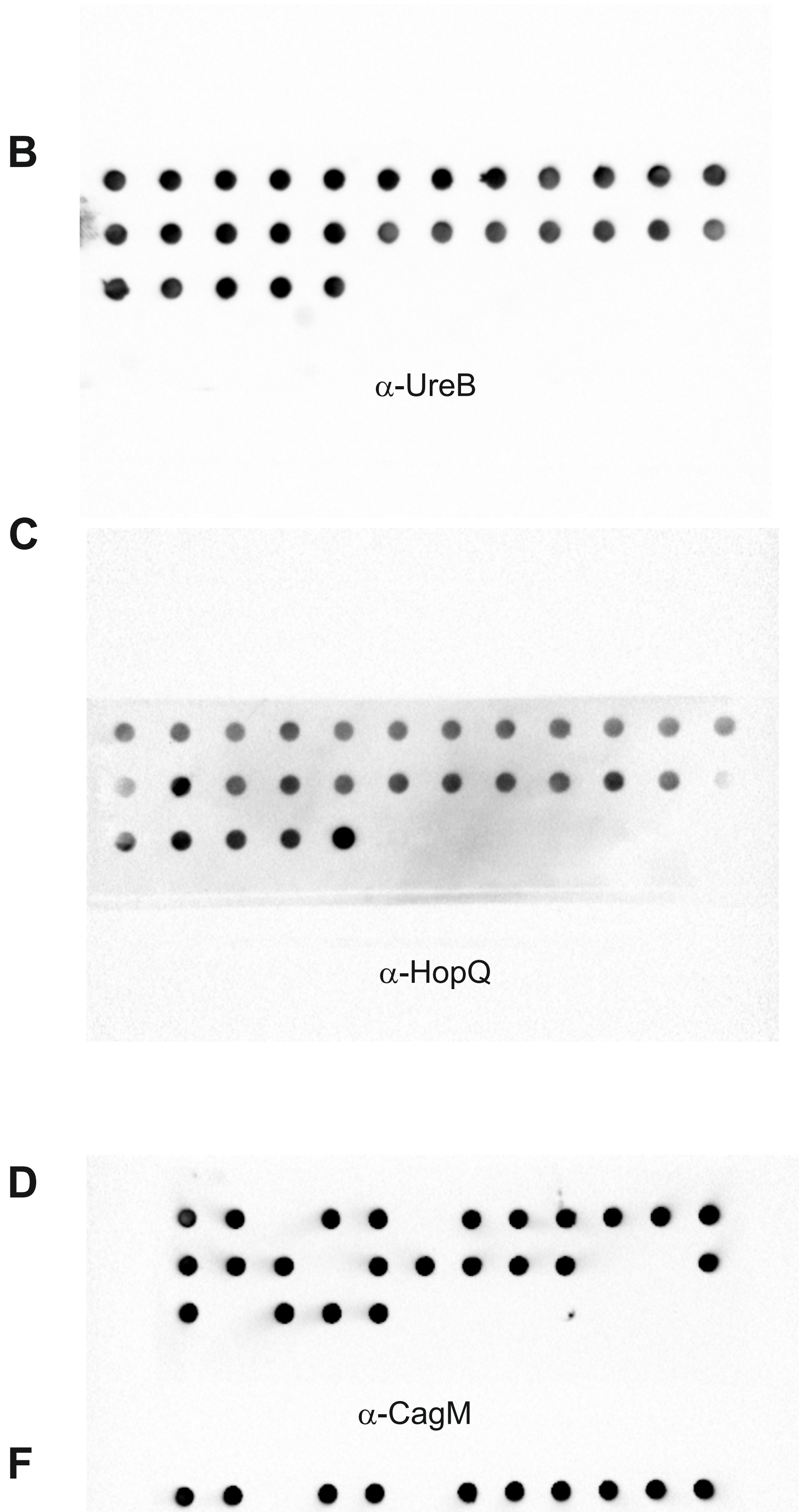

Figure S1 (continued)

E

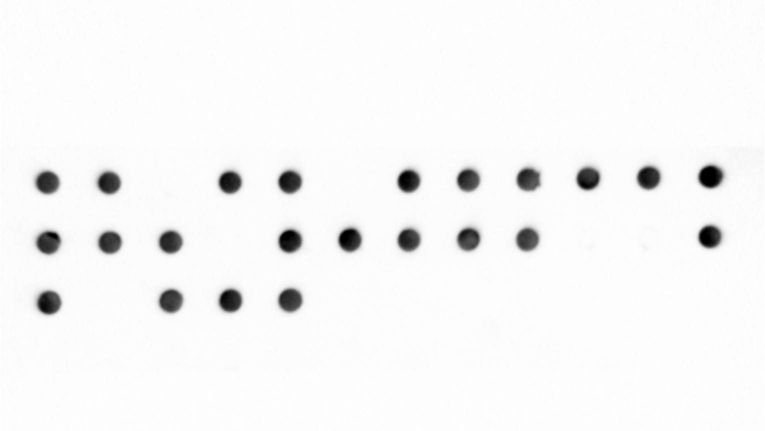

α-CagY

G

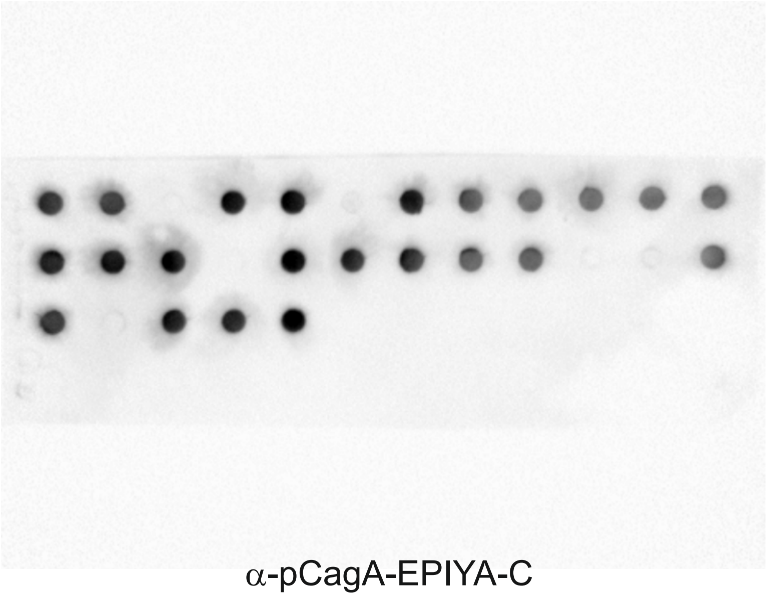

α-pCagA-EPIYA-C
